# A Novel Peptide Targeting the USP25–SIRT1 Interaction Activates SIRT1 to Suppress Cell Senescence

**DOI:** 10.1111/acel.70786

**Published:** 2026-10-30

**Authors:** YuJuan Zhou, Xinshu Wang, Jiyuan Zhang, Hanqiong Qiang, Xinru Zhao, Jingyi Luo, Yanjun Ji, Jiakai Yao, Linhui Zhai, Tingting Zhang, Ke Li, Yuping Chen, Jian Yuan, Lei Li, Yunhui Li

**Affiliations:** ^1^ State Key Laboratory of Cardiovascular Diseases and Medical Innovation Center, Shanghai East Hospital, School of Medicine Tongji University Shanghai China; ^2^ Sanming Hospital of Integrated Medicine Sanming China; ^3^ Department of Biochemistry and Molecular Biology Tongji University School of Medicine Shanghai China; ^4^ Translational Research Institute of Brain and Brain‐Like Intelligence Shanghai Fourth People's Hospital School of Medicine Tongji University Shanghai China; ^5^ State Key Laboratory of Bioactive Substance and Function of Natural Medicines, Institute of Medicinal Biotechnology Chinese Academy of Medical Sciences & Peking Union Medical College Beijing China; ^6^ Cancer Center Tongji University School of Medicine Shanghai China; ^7^ Clinical Center for Brain and Spinal Cord Research Tongji University Shanghai China; ^8^ Affiliated Shanghai Blue Cross Brain Hospital, School of Medicine Tongji University Shanghai China

**Keywords:** aging, cell senescence, cell‐penetrating peptides, SIRT1, USP25

## Abstract

The deacetylase SIRT1 is a critical regulator of cellular senescence, and the excessive accumulation of senescent cells can further drive the onset of organismal aging. SIRT1 activity is tightly governed by posttranslational modifications, including ubiquitination. While ubiquitination has been well documented to control SIRT1 protein stability, the precise regulatory pathway underlying SIRT1 activity remains poorly understood. Here, we identify the deubiquitinase USP25 as a central regulator of cellular senescence. Specifically, USP25 deficiency in mice mitigates age‐related phenotypes and improves overall physiological function. Mechanistically, USP25 directly interacts with SIRT1 and mediates its deubiquitylation via K63‐linked polyubiquitination, specifically targeting SIRT1 lysine residues K506 and K513, which ultimately suppresses SIRT1 deacetylase activity. Reduced SIRT1 deacetylase activity, in turn, increases P53 or PGC1α acetylation, which further increases the transcriptional activation of downstream target genes and consequently accelerates cellular senescence. Furthermore, we developed a cell‐penetrating peptide, pSirt1‐4, that efficiently disrupts the USP25–SIRT1 interaction, increases SIRT1 deacetylase activity, and attenuates cellular senescence. Collectively, our findings establish USP25 as a critical regulator of aging and highlight the USP25–SIRT1 axis as a promising therapeutic target for treating senescence‐related diseases.

## Graphical abstract

USP25‐mediated K63‐linked deubiquitination of SIRT1 at K506 and K513 suppresses its activity to promote senescence. These findings unveil a novel aging regulatory axis and identify a peptide targeting the USP25–SIRT1 interaction for anti‐senescence therapeutic intervention.

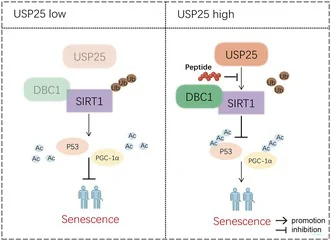

## 1 Introduction

Cellular senescence is a complex biological process that regulates physiological functions, such as embryonic development and wound healing, and contributes to aging, disease, and metabolic disorders (Hernandez‐Segura et al. 2018; Rossiello et al. 2014). Senescent cells exhibit distinct features, including an enlarged, flat morphology, organelle abnormalities, irreversible growth arrest, and a senescence‐associated secretory phenotype (SASP); biomarkers, such as SA‐β‐gal, P16, and P21, are used to identify senescence (Baker et al. 2016; Coppé et al. 2008; Di Micco et al. 2021; Dulić et al. 2000; Kumari and Jat 2021). SIRT1, an NAD^+^‐dependent deacetylase, is a central regulator of aging that counters cellular senescence by modulating key targets such as P53, PGC1α, and FOXOs, increasing genomic stability, and promoting metabolic health (Luo et al. 2000; Nacarelli et al. 2019; van der Horst et al. 2004). Several therapeutic strategies have been developed to harness the antiaging potential of SIRT1, such as activating SIRT1 directly with compounds such as STACs (e.g., SRT1720) or indirectly by increasing NAD^+^ levels with precursors (NMN and NR) or by inhibiting its negative regulators (Childs et al. 2015; Petrova et al. 2016; Xu et al. 2018). However, the majority of SIRT1 activators remain in preclinical stage or early‐stage clinical trials, as the optimal intervention window and dosage for aging treatment remain unclear and their long‐term safety and efficacy have yet to be fully validated.

Protein ubiquitination is a dynamic posttranslational modification that regulates diverse eukaryotic processes, including apoptosis, cell division, and the immune response. Ubiquitination mediates the degradation, interactions, and functional regulation of proteins depending on the type of ubiquitin conjugation (Buetow and Huang 2016; Dikic and Schulman 2023; Park et al. 2021; Swatek and Komander 2016). Ubiquitination is reversible through the actions of deubiquitinating enzymes (DUBs), which modulate substrate protein stability and function to impact biological and pathophysiological processes (Komander et al. 2009; Nijman et al. 2005). When cells enter senescence, protein turnover slows, and factors such as oxidative damage lead to the accumulation of ubiquitinated proteins, resulting in cellular dysfunction. Emerging evidence suggests that DUBs play important and complex regulatory roles in age‐related diseases by modulating protein homeostasis, mitochondrial function, and the cell cycle in senescent cells (Cunningham et al. 2015; Goldbraikh et al. 2020; Kumari et al. 2020; Li et al. 2022).

USP25 is a deubiquitinating enzyme that plays key roles in regulating inflammation, the immune response, and cancer (Valero et al. 1999). As a deubiquitinating enzyme (DUB), USP25 removes polyubiquitin chains from substrate proteins, thereby restoring and stabilizing their biologically active forms. Additionally, USP25 has been implicated in amyloid pathology, tissue injury and cancer therapy (Cai et al. 2023; Li et al. 2024; Wang et al. 2020; Zheng et al. 2022). It mediates the K63‐linked deubiquitination and modification of SHLD2 at specific lysine residues, thereby enhancing the nonhomologous end joining (NHEJ) DNA repair pathway and consequently reducing the chemosensitivity of cancer cells (Li et al. 2024). However, the underlying effects and mechanisms of USP25 in cellular senescence and aging remain unclear.

In this study, we revealed that USP25 plays an important role in the aging process in mice. USP25 exhibits gradually increased protein expression during aging and deubiquitinates SIRT1 to inhibit its deacetylase activity, thereby regulating the transcription of P53 or PGC1α downstream targets and affecting aging. We also designed small‐molecule cell‐penetrating peptides targeting the USP25 and SIRT1 interaction interface and proposed that these peptides may serve as potential antiaging drugs. In summary, our work reveals a novel mechanism through which the USP25–SIRT1 axis acts as a critical aging regulator and suggests its potential as a target for age‐related diseases.

## 2 Results

### 2.1 USP25 Is an Age‐Associated Factor

We previously identified USP25 as a key regulator of DDR and generated the *Usp25* ^−/−^ mice (Li et al. 2024). We serendipitously found that the lifespan of *Usp25* ^−/−^ mice was longer than that of their wild‐type control littermates (Figure 1A). Additionally, compared with wild‐type control littermates, *Usp25* ^−/−^ mice exhibited improved rotarod performance at the ages of 5 and 20 months (Figure 1B). To investigate the potential role of USP25 in aging, we examined the expression of USP25 in aged C57/BL6 mice. Compared with that in the livers of young (5‐month‐old) mice, the protein expression of USP25 in the livers of 20‐month‐old mice significantly increased (Figure 1C,D). However, the mRNA level of USP25 remained unchanged, as revealed by quantitative reverse transcriptase PCR (qRT–PCR) (Figure 1E). Moreover, we investigated the potential role of USP25 in cell senescence by first examining the expression of USP25 in MCF‐10A cells following treatment with doxorubicin. The results revealed that the protein level of USP25, but not the mRNA level of USP25, markedly increased after treatment with doxorubicin (Figure 1F,G). Next, we stably knocked down USP25 expression in MCF‐10A cells using two independent short hairpin RNAs (shRNAs) (Figure 1H) to determine whether USP25 affects doxorubicin‐induced SA‐β‐gal, a widely accepted biomarker that can distinguish senescent cells, upon staining for this marker (Dimri et al. 1995). As shown in Figure 1I,J, doxorubicin‐induced senescent MCF‐10A cells displayed strongly positive SA‐β‐gal staining, whereas USP25‐knockdown cells exhibited less SA‐β‐gal staining. Similarly, the protein level of P21, a well‐recognized senescence marker, significantly decreased in USP25‐knockdown cells (Figure 1K). Additionally, we performed RNA sequencing on liver tissues from 10‐month‐old *Usp25* ^+/+^ and *Usp25* ^−/−^ mice. RNA‐seq‐based clustering analysis revealed highly significant differences in transcriptome between *Usp25* ^−/−^ and *Usp25* ^+/+^ mice, as evidenced by the hierarchical clustering heatmap (Figure 1L). Gene set enrichment analysis (GSEA) revealed significant suppression of inflammatory and apoptotic gene sets in *Usp25* ^−/−^ mice, suggesting that USP25 deficiency effectively curtails apoptotic and inflammatory responses in vivo (Figure 1M; Figure S1A). Collectively, these results suggest that USP25 serves as an age‐associated factor.

**FIGURE 1:**
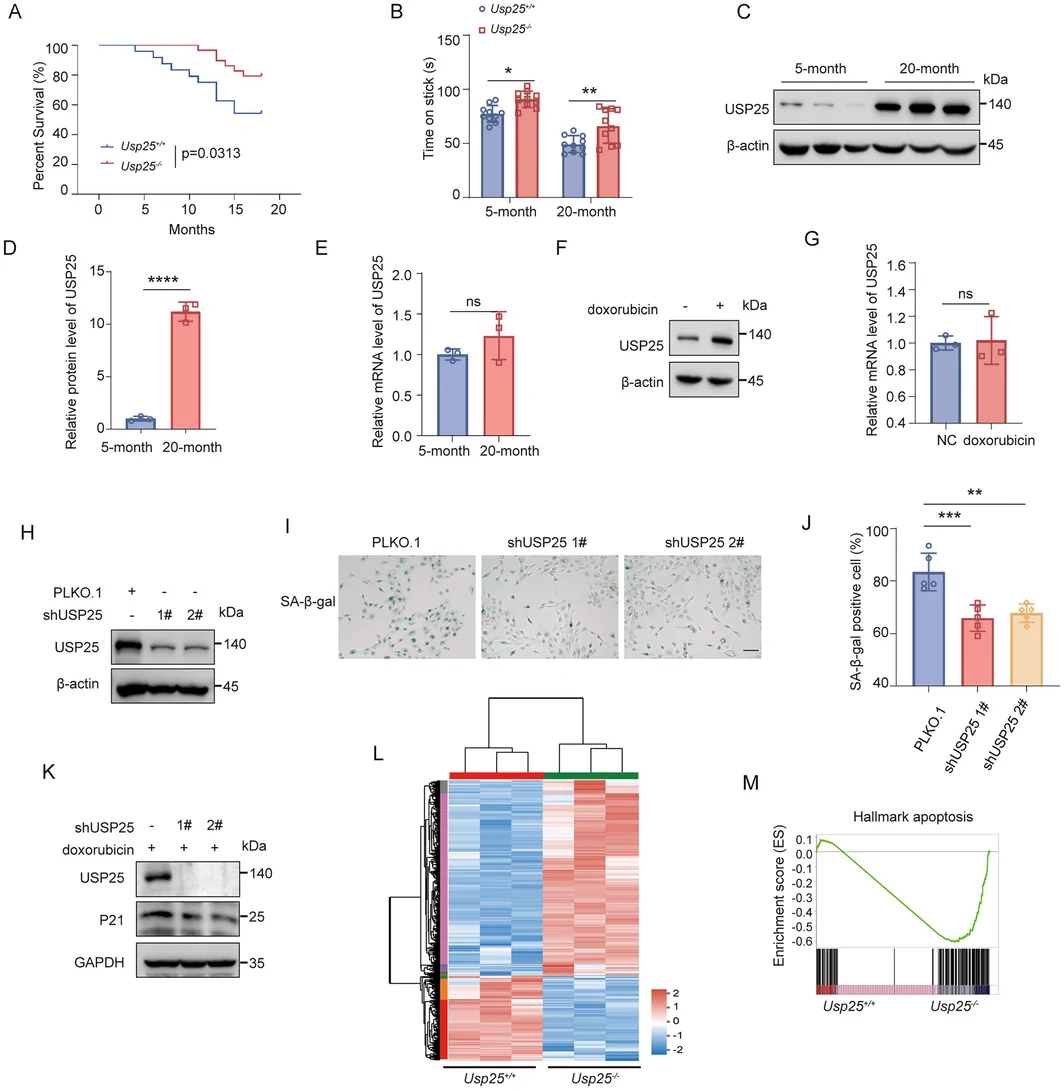
USP25 is preferentially accumulated during aging. (A) Survival curves of *Usp25* ^+/+^ and *Usp25* ^−/−^mice in 20 months (*Usp25* ^+/+^: *n* = 24; *Usp25* ^−/−^: *n* = 29). Log‐rank test (*p* = 0.0313). (B) Effect of aging on the average time to fall off the rotarod. Data are presented as the mean ± SD (*n* = 10 per group). (C) Representative Western blot bands of USP25 and β‐actin in liver tissues from 5‐month‐old and 20‐month‐old mice. (D) Densitometric quantification of USP25 normalized to β‐actin. Data are presented as the mean ± SD (*n* = 3 per group). (E) Fold change in USP25 mRNA expression in aged mice compared with that in the young mice in liver tissue. Data are presented as the mean ± SD (*n* = 3 per group). (F) The relative protein levels of USP25 in MCF‐10A cells treated with doxorubicin for 24 h. (G) The relative mRNA expression levels of USP25 were normalized to those of GAPDH levels. Data are presented as the mean ± SD (*n* = 3 per group). (H) MCF‐10A cells were infected with the indicated short hairpin RNA (shRNA) against USP25 and subjected to Western blotting assay. (I) Representative images of SA‐β‐gal staining in control and USP25‐depleted MCF‐10A cells treated with doxorubicin (0.2 μM for 24 h). Scale bar = 100 μm. (J) Quantification of SA‐β‐gal‐positive cells. The percentage of blue‐stained positive cells was calculated by counting total cells and SA‐β‐gal‐positive cells in 5 random bright‐field fields per sample. Data are presented as the mean ± SD. (K) P21 protein levels in USP25 knockdown MCF‐10A cells treated with doxorubicin were determined by immunoblotting. (L) Distinct differential gene expression heatmaps for 10‐month‐old *Usp25* ^+/+^ and *Usp25* ^−/−^ mice (*n* = 3 per group). (M) GSEA of RNA sequencing data from 10‐month‐old *Usp25* ^+/+^ and *Usp25* ^−/−^ mice (*n* = 3 per group). Statistical analysis was performed via two‐way ANOVA (B), *t*‐tests (D, E, and G) or one‐way ANOVA (J), *p ≤ 0.05, **p ≤ 0.01, ***p ≤ 0.001, ****p ≤ 0.0001; ns indicates no statistically significant difference.

### 2.2 USP25 Deletion Alleviates Aging Phenotypes in Mice

An increased number of senescent cells contributes to aging and age‐related pathologies (Muñoz‐Espín and Serrano 2014); therefore, we evaluated the physiological and pathological roles of USP25 during aging in vivo. We monitored *Usp25* ^−/−^ mice and their wild‐type control littermates (*Usp25* ^+/+^) until they reached 10 months of age. Compared with *Usp25* ^+/+^ mice, 10‐month‐old *Usp25* ^−/−^ mice had a lower body weight (Figure 2A), and exhibited a lower liver‐to‐body weight ratio (Figure 2B). To further investigate the molecular basis of these phenotypic changes, we found that the protein expression of P21 was significantly lower in the lungs of 10‐month‐old *Usp25* ^−/−^ mice than in control mice and that the mRNA levels of P16 and P21 were consistently altered (Figure 2C–F). In addition, P21 staining revealed P21 expression was weaker in liver samples from *Usp25* ^−/−^ mice than from *Usp25* ^+/+^ mice (Figure 2G,H). Consistent with these liver findings, we also performed H&E staining and P21 immunohistochemistry on lung, brain, heart, and kidney tissues from *Usp25* ^−/−^ and *Usp25* ^+/+^ mice. Usp25 deficiency markedly reduced P21 expression in these organs (Figure S1B–D), further indicating that Usp25 knockout effectively ameliorated the aging phenotype in mice.

**FIGURE 2:**
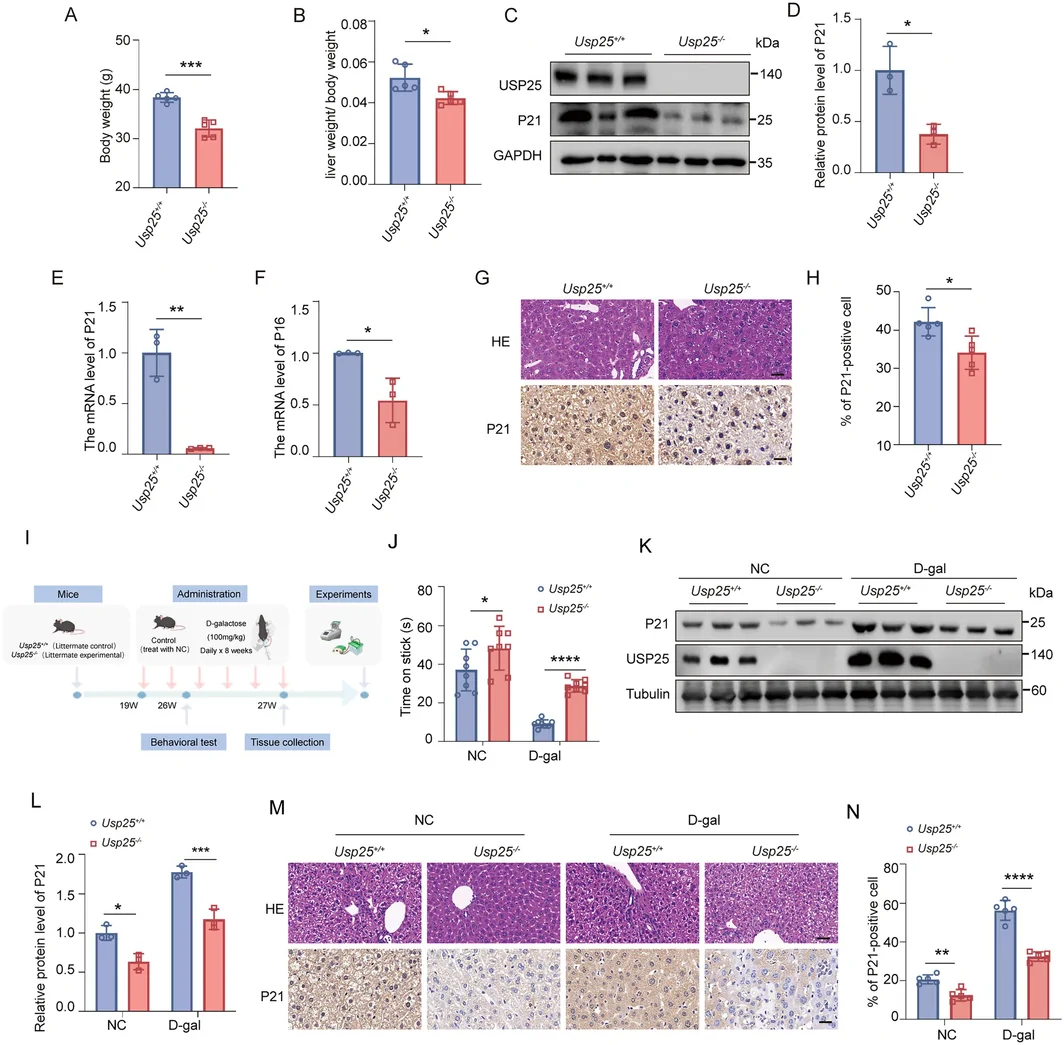
USP25 deficiency alleviates aging phenotypes and D‐galactose‐induced aging in vivo. (A) Body weight of *Usp25* ^+/+^ and *Usp25* ^−/−^ mice. Data are presented as the mean ± SD (*n* = 5 per group). (B) Liver‐to‐body weight ratios of *Usp25* ^+/+^ and *Usp25* ^−/−^ mice. Data are presented as the mean ± SD (*n* = 5 per group). (C) P21 protein level in 10‐month‐old *Usp25* ^+/+^ and *Usp25* ^−/−^ mice. (D) Quantification of P21 normalized to that of GAPDH. Data are presented as the mean ± SD (*n* = 3 per group). (E, F) Fold changes in P21 mRNA (E) and P16 mRNA (F) in the lung of 10‐month‐old *Usp25* ^+/+^ and *Usp25* ^−/−^ mice in liver tissue. Data are presented as the mean ± SD (*n* = 3 per group). (G) Representative H&E (scale bar = 50 μm) and P21 (scale bar = 20 μm) staining of the liver of *Usp25* ^+/+^ and *Usp25* ^−/−^ mice. (H) Quantification of P21 staining in the livers of *Usp25* ^+/+^ and *Usp25* ^−/−^ mice. Data are presented as the mean ± SD (*n* = 5 per group). (I) Schematic models illustrating the D‐galactose‐induced mouse model. (J) Comparison of the average time to fall off the rotarod among the indicated groups of mice. Data are presented as the mean ± SD (*n* = 8 per group). (K) P21 protein levels in liver lysates from *Usp25* ^+/+^ and *Usp25* ^−/−^ of both the untreated conditions and after D‐gal treatment. (L) Quantification of P21 normalized to tubulin. Data are presented as the mean ± SD (*n* = 3 per group). (M) Representative H&E (scale bar = 50 μm) and P21 (scale bar = 20 μm) staining of the liver. (N) Quantification P21 staining of the liver of *Usp25* ^+/+^ and *Usp25* ^−/−^ mice. Data are presented as the mean ± SD (*n* = 5 per group). Statistical analysis was performed via *t*‐tests (A, B, D, E, F, and H) or two‐way ANOVA (J, L, and N), *p ≤ 0.05, **p ≤ 0.01, ***p ≤ 0.001, ****p ≤ 0.0001.

Aging can directly affect the differentiation and function of osteoblasts; therefore, we assessed osteoclast and osteoblast activity via tartrate‐resistant acid phosphatase TRAP (a marker of osteoclasts) and alkaline phosphatase ALP (a marker of osteoblasts) staining. As shown in Figure S1E–G, compared with their wild‐type control littermates, *Usp25* ^−/−^ mice presented significantly increased ALP^+^ osteoblast numbers and notably fewer TRAP^+^ osteoclasts, indicating enhanced bone formation and suppressed bone resorption in *Usp25* ^−/−^ mice. Thus, the aging‐related bone loss phenotype was alleviated. Taken together, these data suggest that USP25 deletion alleviates aging phenotypes in mice, indicating that USP25 may be a causal factor in aging.

### 2.3 USP25 Deficiency Ameliorates D‐Galactose‐Induced Aging in Mice

The D‐galactose (D‐gal)‐induced mouse model, a well‐established accelerated aging tool used for nearly two decades, recapitulates major aging hallmarks such as cognitive impairment, chronic inflammation and cellular senescence in multiple tissues and organs (Sahu et al. 2024; Vaseenon et al. 2025). To further elucidate the role of USP25 in murine aging, we assigned *Usp25* ^+/+^ and *Usp25* ^*−/*−^ mice to either a D‐gal‐treated group (100 mg/kg, i.p., daily for 8 weeks; Figure 2I) or a normal control group (NC). Rotarod tests revealed that there was a difference in the rotarod performance between *Usp25* ^+/+^ and *Usp25* ^*−/*−^ mice. In both the untreated conditions and after D‐gal treatment, *Usp25* ^−/−^ mice stayed on the rotarod for a longer time than their wild‐type littermate controls did (Figure 2J). Furthermore, the Western blot results demonstrated that the protein expression level of P21 in *Usp25* ^−/−^ mice was significantly lower than that in *Usp25* ^+/+^ mice in both the untreated and D‐gal‐treated groups (Figure 2K,L). Similarly, P21 staining revealed that USP25 knockout mice reduced P21 accumulation in liver samples after D‐gal treatment (Figure 2M,N). We also evaluated osteoclast/osteoblast activity. In both the untreated conditions and after D‐gal treatment, *Usp25* ^−/−^ mice had significantly more ALP^+^ osteoblasts and notably fewer TRAP^+^ osteoclasts (Figure S2A,B). Collectively, these results suggest that USP25 knockout ameliorates D‐galactose‐induced aging, pointing to a potentially important role for USP25 in aging.

### 2.4 USP25 Directly Interacts With and Deubiquitinates SIRT1 Through K63‐Linked Ubiquitination

To explore the mechanism by which USP25 regulates cellular senescence and organismal aging, we performed mass spectrometry analysis, which revealed multiple USP25 interacting proteins, including SIRT1, a newly discovered interactor (Figure 3A). As a deacetylase, SIRT1 acts as a key regulator of both cellular senescence and aging. We subsequently confirmed the interaction between USP25 and SIRT1 via coimmunoprecipitation (co‐IP) assays in HEK‐293 T cells (Figure 3B,C). We next sought to determine whether USP25 modulates the protein expression of SIRT1. Western blot analysis revealed that depletion of USP25 did not significantly influence SIRT1 protein levels (Figure 3D). Consistent with these results, neither overexpression of wild‐type USP25 (USP25^WT^) nor the catalytically inactive mutant (USP25^C178S^) altered SIRT1 protein expression (Figure 3E). Moreover, we validated these findings in mouse tissues, confirming that USP25 knockout does not affect SIRT1 levels in vivo (Figure S3A).

**FIGURE 3:**
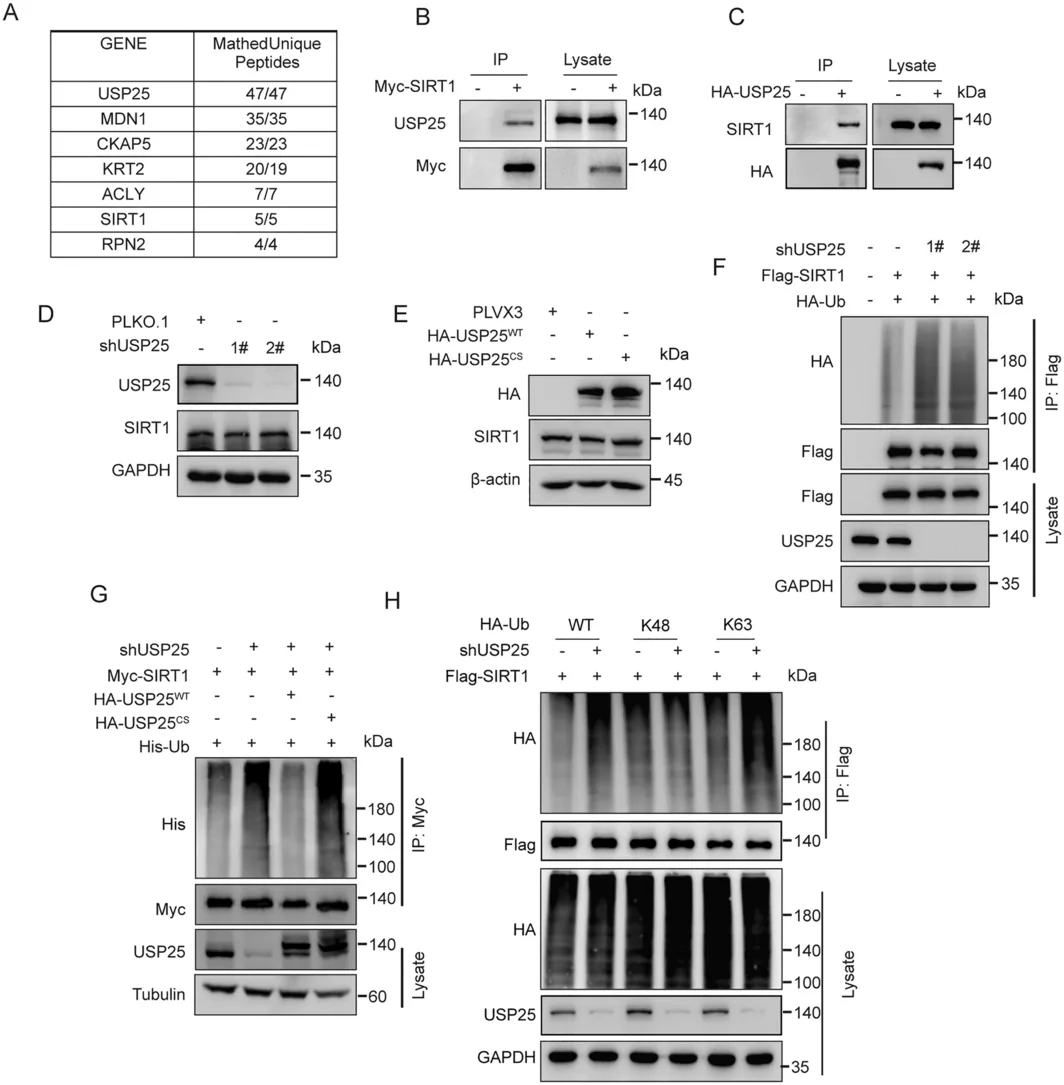
USP25 directly interacts with and deubiquitinates SIRT1. (A) IP–MS analysis was performed to screen for USP25‐interacting proteins. A partial list of accession numbers and the matched peptides of candidate proteins is shown. (B) Anti‐Myc immunoprecipitation was performed in HEK‐293 T cells transfected with Myc‐SIRT1, and the precipitates were immunoblotted for USP25. (C) Anti‐HA immunoprecipitation was performed in HEK‐293 T cells transfected with HA‐USP25, and the precipitates were immunoblotted for SIRT1. (D) The protein levels of SIRT1 in USP25 stable knockout cells were determined by immunoblotting. (E) The protein levels of SIRT1 were determined by immunoblotting under conditions in which USP25^WT^ or catalytically inactive USP25^C178S^ was overexpressed. (F) USP25‐knockdown cells transfected with Flag‐SIRT1 and HA‐Ub were lysed and immunoprecipitated with anti‐Flag. The immunoprecipitates were blotted for HA and Flag to measure the ubiquitination level of SIRT1. (G) USP25‐knockdown cells were transfected with Myc‐SIRT1, His‐Ub, and HA‐USP25 (WT or C178S) and then subjected to anti‐Myc IP and immunoblotting for His to detect the ubiquitination level of SIRT1. (H) USP25‐knockdown and control HEK‐293 T cells were transfected with Flag‐SIRT1 and HA‐Ub (WT, K48, or K63), and then subjected to anti‐Flag IP and immunoblotting for HA to detect the level of ubiquitinated SIRT1.

Given that USP25 is a typical deubiquitinase, we further explored whether USP25 regulates SIRT1 through its deubiquitination activity. Ubiquitination assays revealed that knockdown of USP25 led to a significant increase in SIRT1 ubiquitination levels (Figure 3F). In contrast, overexpression of USP25^WT^, but not the catalytically inactive USP25^C178S^ mutant, reduced SIRT1 ubiquitination (Figure 3G). Meanwhile, we examined endogenous SIRT1 ubiquitination in tissues from *Usp25* ^+/+^ and *Usp25* ^−/−^ mice. Consistent with the above results, SIRT1 ubiquitination levels were increased in *Usp25* ^−/−^ mice (Figure S3B). K48 and K63 ubiquitination chains are the two most common types of polyubiquitination, accounting for 52% and 38% of ubiquitination events, respectively (Dammer et al. 2011). Specifically, K48 chains target substrates for 26S proteasomal degradation and are critical for proteolysis, whereas K63 chains mainly regulate protein activity, localization, and signaling (Chau et al. 1989; Komander and Rape 2012). As USP25 did not affect SIRT1 protein levels, we sought to investigate whether USP25 can facilitate the K63‐linked ubiquitination of SIRT1. The results demonstrated that knockdown of USP25 specifically increased the level of K63‐linked polyubiquitin chains on SIRT1, but not K48‐linked polyubiquitin chains (Figure 3H). In conclusion, these data revealed that USP25 directly binds to SIRT1 and mediates its K63‐linked deubiquitination to regulate its activity without affecting its degradation.

### 2.5 USP25 Deletion Suppresses Cellular Senescence by Modulating SIRT1‐Mediated P53 Acetylation and Transcriptional Activity

SIRT1 is an NAD‐dependent deacetylase and a core aging regulator. It antagonizes cellular senescence by modulating pivotal downstream targets (Luo et al. 2000; Nacarelli et al. 2019; van der Horst et al. 2004). On the basis of our findings that the deubiquitinase USP25 is a central regulator of cellular senescence and aging, we hypothesized that USP25 modulates senescence through the regulation of SIRT1 deacetylase activity. First, we constructed stable USP25‐knockdown cells and reported that USP25 depletion significantly increased cellular SIRT1 deacetylase activity (Figure 4A,B; Figure S4A,B). Consistent with these findings, USP25 knockout significantly increased SIRT1 deacetylase activity in vivo (Figure S4C). Moreover, we established USP25 and SIRT1 single‐knockdown and double‐knockdown MCF‐10A cell lines to determine whether the regulatory effect of USP25 on cellular senescence is dependent on SIRT1 (Figure 4C). After treatment with doxorubicin, USP25 knockdown reduced the number of SA‐β‐gal positive cells, whereas SIRT1 knockdown increased SA‐β‐gal activity. However, further depletion of USP25 in SIRT1‐knockdown cells did not affect SA‐β‐gal activity (Figure 4D,E). Collectively, these results demonstrated that USP25 regulates cellular senescence in a SIRT1‐dependent manner.

**FIGURE 4:**
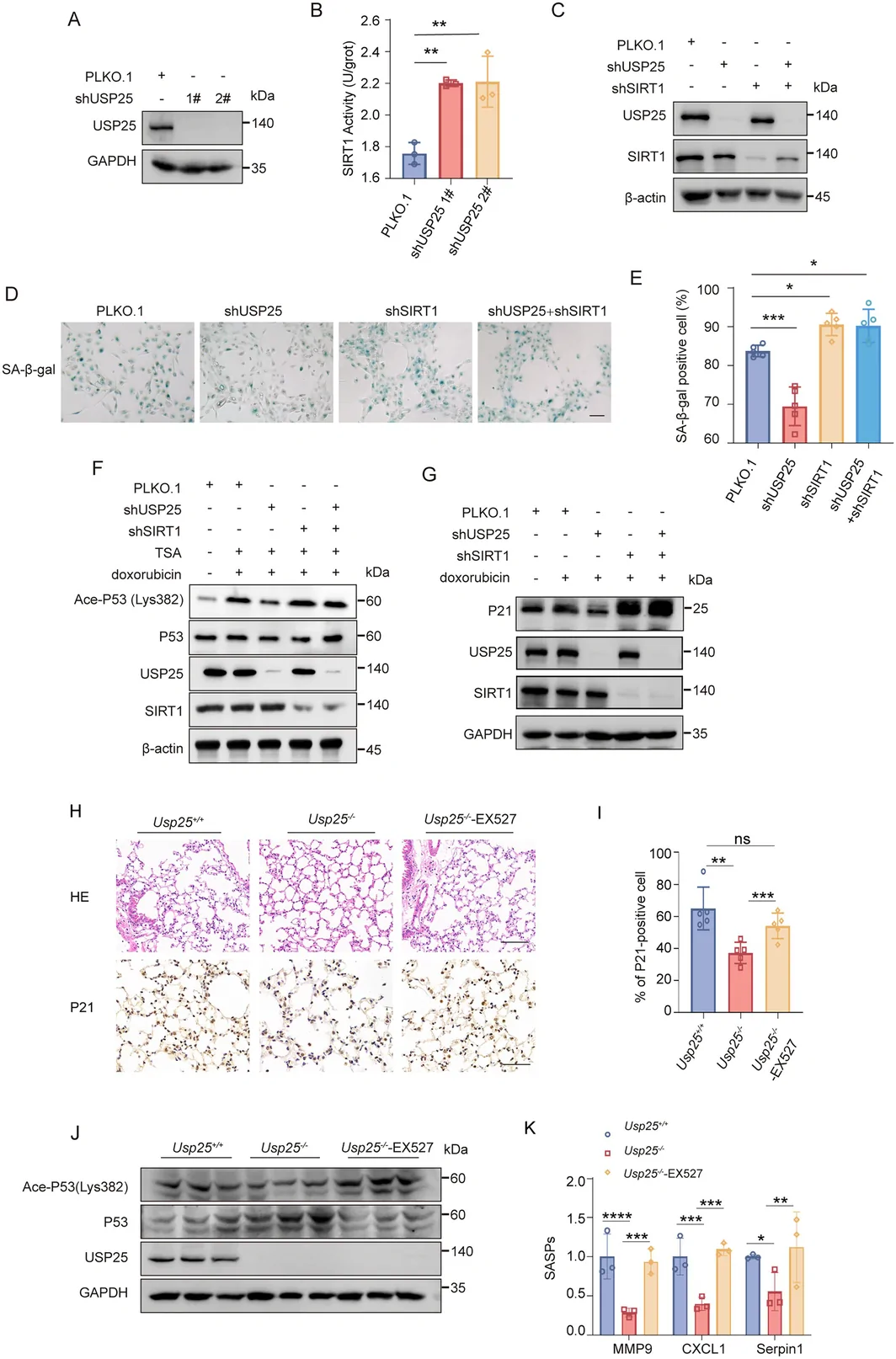
USP25 deletion suppresses cell senescence through SIRT1‐mediated P53 acetylation. (A) Immunoblot of USP25 in the MCF‐10A cells stably expressing control and USP25shRNA. (B) ELISA analysis of SIRT1 deacetylase activity in USP25‐knockdown MCF‐10A cells. The data are presented as the mean ± SD (*n* = 3 per group). (C) Immunoblotting was performed to detect USP25 and SIRT1 expression in stably expressing MCF‐10A cells. (D) Representative images of SA‐β‐gal staining in the indicated MCF‐10A cell lines. Scale bar = 100 μm. (E) Quantitative analysis of senescence‐associated β‐galactosidase (SA‐β‐gal) staining in the indicated MCF‐10A cell lines. The data are presented as the mean ± SD (*n* = 5 per group). (F) Western blot analysis of Ace‐P53(Lys382) in the indicated MCF‐10A cells. (G) Immunoblotting of P21 was shown from USP25 and SIRT1 knockdown MCF‐10A cells. (H) Representative H&E (scale bar = 100 μm) and P21 (scale bar = 50 μm) staining of the lungs of *Usp25* ^+/+^, *Usp25* ^−/−^ mice, and *Usp25* ^−/−^ mice treated with EX527. (I) Quantification of P21 staining of the lungs of *Usp25* ^+/+^, *Usp25* ^−/−^ mice, and *Usp25* ^−/−^ mice treated with EX527. The data are presented as the mean ± SD (*n* = 5 per group). (J) Ace‐P53 protein levels in lung lysates from *Usp25* ^+/+^ mice, *Usp25* ^−/−^ mice, and *Usp25* ^−/−^ mice treated with EX527. (K) Relative expression of MMP9, CXCL1, and Serpin1 mRNA. The data are presented as the mean ± SD (*n* = 3 per group). Statistical analysis was performed via one‐way ANOVA (B, E, and I) or two‐way ANOVA (K), *p ≤ 0.05, **p ≤ 0.01, ***p ≤ 0.001.

As several studies have demonstrated that reduced SIRT1 deacetylase activity increases the acetylation of P53 or PGC1α and thereby accelerates cellular senescence, we assessed whether USP25 depletion inhibits the acetylation of SIRT1 downstream targets. As shown in Figure 4F and Figure S4D–G, USP25 knockdown significantly inhibited P53 acetylation at K382 and PGC‐1α acetylation, but failed to suppress P53 and PGC‐1α acetylation in USP25‐ and SIRT1‐double‐knockdown cells. These results clearly indicate that USP25‐mediated suppression of P53 or PGC‐1α acetylation depends on SIRT1. To support this conclusion, we further demonstrated that USP25 knockdown, but not USP25‐ and SIRT1‐double‐knockdown cells, reduced P21 levels (Figure 4G). These results demonstrated that USP25 regulates p53 or PGC‐1α acetylation and P21 levels in a SIRT1‐dependent manner.

To further determine whether the antiaging effect of USP25 deficiency is SIRT1–p53 dependent in vivo, we treated *Usp25* ^*−/−*^ mice with EX527, a SIRT1‐specific inhibitor, in a D‐gal‐induced aging model. Immunohistochemical (IHC) staining unveiled that the proportion of P21‐positive cells in lung tissue was significantly lower in *Usp25* ^*−/−*^mice than in *Usp25* ^+/+^ mice treated with D‐gal, and EX527 treatment significantly reversed this decrease (Figure 4H,I). In parallel, Western blot analysis revealed that Ace‐P53(Lys382) levels were markedly reduced in *Usp25* ^*−/−*^ mice, but restored by EX527 (Figure 4J). Furthermore, the mRNA levels of SASP‐related factors, including MMP9, CXCL1, and Serpine1, were significantly decreased in *Usp25* ^*−/−*^ mice, whereas EX527 reversed this suppression (Figure 4K). Taken together, these in vivo results indicate that USP25 deficiency delays cellular senescence and inhibits SASPs in a SIRT1–P53 dependent manner.

### 2.6 K506 and K513 Are the Major Sites of SIRT1 Ubiquitination

Ubiquitination begins with the covalent linkage of a single ubiquitin molecule to specific lysine residues on the target substrate. To identify potential ubiquitination sites in SIRT1, we performed mass spectrometry (MS) (Swatek and Komander 2016). Two sites of SIRT1 (K506 and K513) were identified as possible ubiquitination sites (Figure 5A,B). We also found that K506 and K513 are conserved in SIRT1 sequences from humans, house mice, brown rats, goats, cattle, horses, and European rabbits (Figure 5C). To further determine whether these two lysine residues are the primary sites of SIRT1 ubiquitination, we generated a SIRT1 mutant in which these two lysine residues were replaced with arginine residues (SIRT1^2KR^). Our data revealed that the loss of USP25 dramatically increased the ubiquitination of wild‐type SIRT1 but not that of the SIRT1^2KR^ mutant (Figure 5D). These results suggest that K506 and K513 are the primary deubiquitination sites on SIRT1 that are regulated by USP25.

**FIGURE 5:**
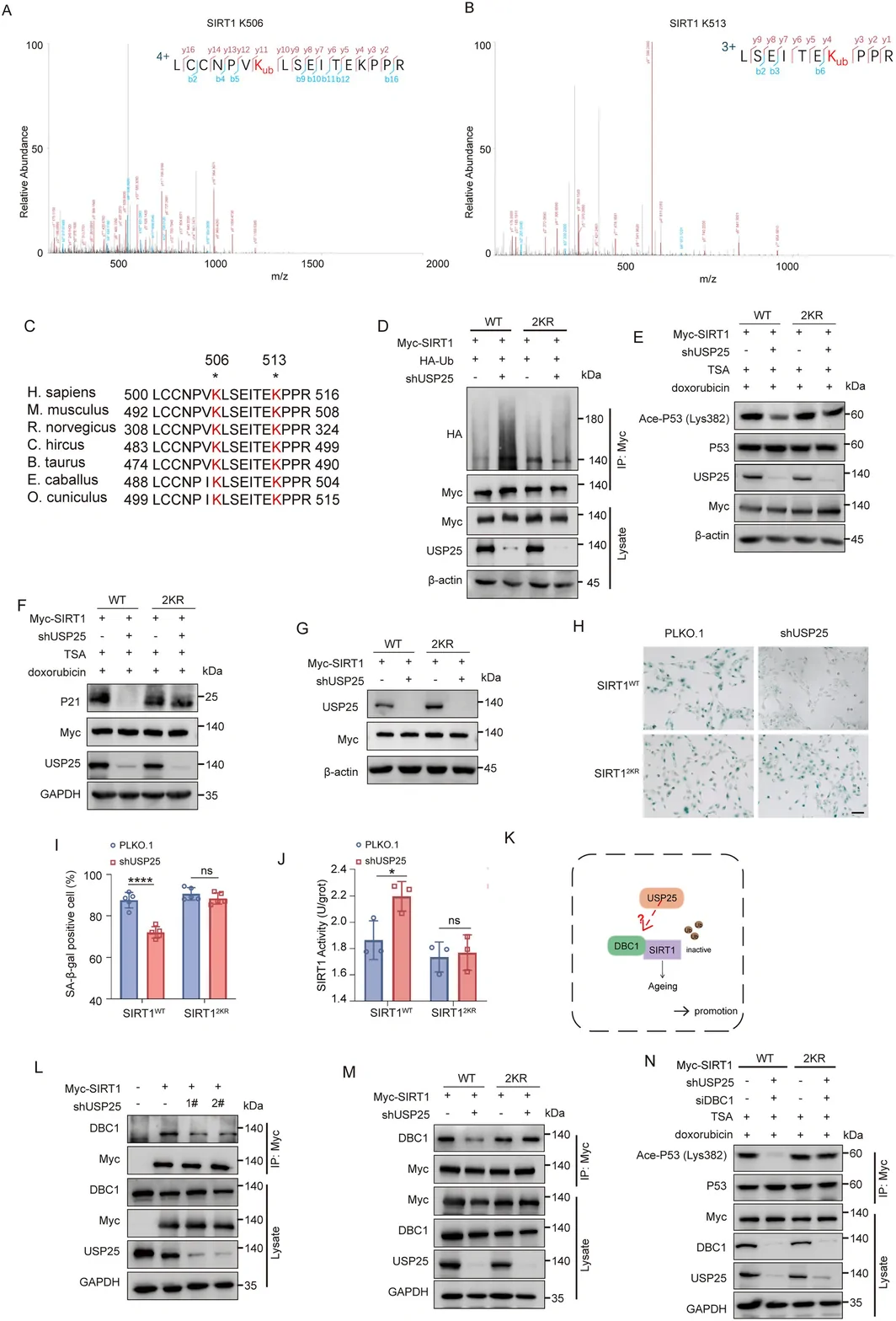
USP25 deubiquitinates SIRT1 at K506/K513 and modulates its deacetylase activity through DBC1. (A, B) Mass spectra identifying ubiquitination at K506 and K513 of SIRT1. (C) The amino acid sequences of SIRT1K506 K513 in humans, house mice, brown rats, goats, cattle, horses, and European rabbits. (D) Control and shUSP25 cells were transfected with SIRT1^WT^ or SIRT1^2KR^ plasmids. After 48 h, the cell lysates were immunoprecipitated with an anti‐Myc antibody. Blots were probed with the indicated antibodies. (E–J) Control or USP25 knockdown cells were transfected with Myc‐SIRT1 or 2KR mutants and subjected to P53 acetylation (E), P21 expression (F), SA‐β‐gal staining assay (*n* = 5 per group) (G–I), and SIRT1 deacetylase activity (*n* = 3 per group) (J). (K) Schematic model illustrating the interplay between USP25, SIRT1, and DBC1. (L) Coimmunoprecipitation (Co‐IP) assays were performed with an anti‐Myc antibody in Myc‐SIRT1‐expressing cells subjected to USP25 knockdown, followed by immunoblotting with the indicated antibodies. (M) Control or USP25 knockdown cells were transfected with Myc‐SIRT1 WT or 2KR mutants and the interaction between DBC1 and SIRT1 was analyzed. (N) Control or USP25‐DBC1 double knockdown cells were transfected with Myc‐SIRT1 or 2KR mutants and the expression of Ace‐P53(Lys382) was determined. Statistical analysis was performed via two‐way ANOVA (I and J), *p ≤ 0.05, ****p ≤ 0.0001; ns indicates no statistically significant difference.

Next, we investigated the functional significance of SIRT1 deubiquitination at K506 and K513 in response to the induction of cellular senescence. As expected, USP25 knockdown suppressed P53 acetylation and P21 expression in the SIRT1^WT^ group but not in the SIRT1^2KR^ mutant group (Figure 5E,F). This trend was consistently observed for the results of the number of SA‐β‐gal‐positive cells (Figure 5G–I). Furthermore, we found that USP25 depletion increased SIRT1 deacetylase activity in wild‐type (WT) SIRT1 but had no effect on the SIRT1^2KR^ mutant in either MCF‐10A (Figure 5J) or HEK‐293 T cells (Figure S5A,B). Taken together, these data indicate that the ubiquitination of SIRT1 at K506 and K513 is critical for the deacetylase activity of SIRT1 and for enhancing the anticell senescence role of SIRT1.

### 2.7 USP25 Modulates SIRT1 Deacetylase Activity Through DBC1

Deleted in breast cancer 1 (DBC1), a nuclear protein with pleiotropic functions, is critically involved in aging and a spectrum of cellular processes (Chini et al. 2013). Prior studies have firmly established that the nuclear protein DBC1 directly binds to SIRT1 to suppress its deacetylase activity. This interaction disrupts the SIRT1‐mediated deacetylation of its downstream substrates (e.g., P53), thereby impairing the ability of SIRT1 to regulate key aging‐associated signaling pathways, including those governing cellular senescence, metabolic homeostasis, and oxidative stress responses (Kang et al. 2011; Kim et al. 2008; Zhao et al. 2008). We therefore investigated whether USP25‐mediated deubiquitination of SIRT1 modulates the DBC1–SIRT1 interaction (Figure 5K). Co‐IP assays demonstrated that USP25 knockdown impaired the binding between SIRT1 and DBC1 (Figure 5L). Moreover, we examined the endogenous interaction between SIRT1 and DBC1 in tissues from *Usp25* ^+/+^ and *Usp25* ^−/−^ mice. Consistent with the above results, the SIRT1–DBC1 interaction was reduced in tissues from *Usp25* ^−/−^ mice (Figure S5C). We found that USP25 depletion decreased the interaction between SIRT1 and DBC1 in the SIRT1^WT^ group, whereas this interaction was unaffected in the SIRT1^2KR^ mutant group (Figure 5M). Furthermore, our results revealed that P53 acetylation decreased in USP25/DBC1 double‐knockdown cells rescued with SIRT1^WT^, but not in those rescued with SIRT1^2KR^ mutant (Figure 5N). These findings indicate that ubiquitination at the K506 and K513 residues of SIRT1 is critical for attenuating its interaction with DBC1.

### 2.8 Peptide‐Mediated Competitive Inhibition of the USP25–SIRT1 Interaction Enhances SIRT1 Deacetylase Activity and Ameliorates Aging Phenotypes

Given that USP25 and SIRT1 orchestrate multiple cellular processes, pharmacological inhibition of these proteins may trigger off‐target perturbations across additional signaling cascades (Ungurianu et al. 2023; Zhou et al. 2023), therefore, strategies that specifically engage the USP25–SIRT1 axis to increase SIRT1 deacetylase activity and suppress cell senescence are urgently needed. Recently, peptides have emerged as promising and specific tools for modulating protein interaction. On the basis of the protein–protein interaction mechanism, multiple strategies have been developed utilizing cell‐penetrating peptides (CPPs) derived from α‐helical sequences to disrupt such protein interactions and block signal transduction (Zhu et al. 2021). To identify the SIRT1 region responsible for USP25 binding, we first generated a panel of SIRT1 truncation mutants (Figure 6A). Mapping revealed that the coiled‐coil‐like (CC‐like) domain of SIRT1 is indispensable for its interaction with USP25 (Figure 6B). Next, we screened peptides targeting this CC‐like domain of SIRT1 by using the predictive I‐TASSER server to analyze its structure. Surface plasmon resonance (SPR) analysis further demonstrated that four peptides (Sirt1‐1, Sirt1‐2, Sirt1‐3, and Sirt1‐4) had high binding affinities for USP25 (Figure 6C, Figure S6A–D).

**FIGURE 6:**
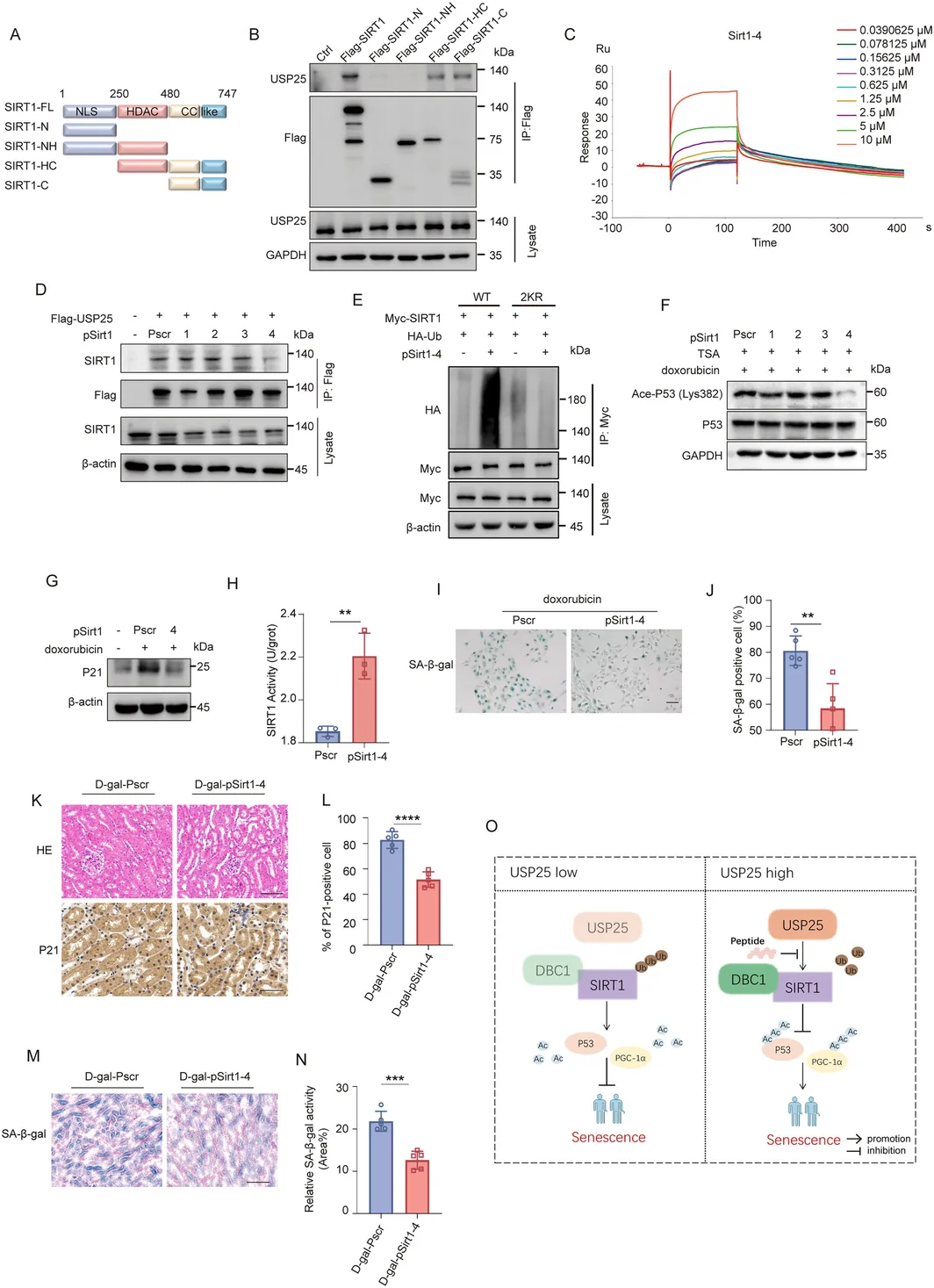
Blocking the USP25–SIRT1 interaction by peptide competition enhances SIRT1 deacetylase activity. (A) Schematic diagram of the truncated SIRT1 protein fragments used for molecular mapping assays. (B) HEK293 T cells were transfected with the truncated SIRT1 protein fragments after which they were subjected to anti‐Flag, and the blots were probed with the indicated antibodies. (C) The kinetic interaction between Sirt1‐4 and USP25 was assessed using surface plasmon resonance (SPR) analyses. (D) Co‐IP assays were performed to detect the interaction between USP25 and SIRT1 in HEK‐293 T cells treated with the indicated peptides. (E) Western blot analysis of levels of the ubiquitination of SIRT1 after treatment with the pSirt1‐4. (F, G) Western blot analysis of the levels of P53 acetylation (F) and P21 (G) was performed in doxorubicin‐stimulated MCF‐10A cells after treatment with the peptides. (H) ELISA analysis of SIRT1 deacetylase activity was detected after pSirt1‐4 treatment in MCF‐10A cells. The data are presented as the mean ± SD (*n* = 3 per group). (I) SA‐β‐gal staining assays were performed after pSirt1‐4 treatment in MCF‐10A cells. Scale bar = 100 μm. (J) Quantitative SA‐β‐gal staining assays were performed after pSirt1‐4 treatment in MCF‐10A cells. The data are presented as the mean ± SD (*n* = 5 per group). (K) Representative H&E (scale bar = 10 μm) and P21 (scale bar = 50 μm) staining of pSirt1‐4 treated D‐gal mice. (L) Quantification of P21 staining was performed. The data are presented as the mean ± SD (*n* = 5 per group). (M) SA‐β‐gal staining assays were performed after pSirt1‐4 treatment in D‐gal mice. Scale bar = 100 μm. (N) Quantification of SA‐β‐gal‐positive area. The data are presented as the mean ± SD (*n* = 5 per group). (O) USP25 maintains SIRT1 deacetylase activity through deubiquitylation, while cell‐penetrating peptides can interfere with the binding of DBC1 to SIRT1, thereby affecting the deacetylation of P53 by SIRT1; when the acetylation level of P53 changes, it ultimately regulates the cellular senescence phenotype. Statistical analysis was performed via *t*‐tests (H, J, L, and N), **p ≤ 0.01, ***p ≤ 0.001, ****p ≤ 0.0001.

To further explore the functions of these peptides, we designed and synthesized four cell‐penetrating peptides (pSirt1‐1, pSirt1‐2, pSirt1‐3, and pSirt1‐4) (Figure S6E). When only pSirt1‐4 was tested, the endogenous USP25–SIRT1 interaction was effectively disrupted, whereas pSirt1‐1, pSirt1‐2, and pSirt1‐3 lacked such inhibitory activity (Figure 6D). Moreover, we assessed the effect of pSirt1‐4 on SIRT1 ubiquitination. pSirt1‐4 markedly enhanced SIRT1 ubiquitination in cells expressing SIRT1^WT^, but not in those expressing the SIRT1^2KR^ mutant (Figure 6E). We next evaluated the biological impact of pSirt1‐4 in doxorubicin‐induced MCF‐10A cells. Notably, pSirt1‐4 treatment significantly reduced P53 acetylation levels and downregulated P21 expression in doxorubicin‐stimulated cells (Figure 6F,G). Consistent with these findings, SIRT1 deacetylase activity assays demonstrated that pSirt1‐4 markedly increased SIRT1 deacetylase activity in both MCF‐10A and HEK‐293 T cells (Figure 6H; Figure S6F). In addition, pSirt1‐4 significantly attenuated SA‐β‐gal activity in doxorubicin‐induced senescent MCF‐10A cells (Figure 6I,J).

To confirm the ability of the pSirt1‐4 peptide to suppress senescence in vivo, we performed in vivo intervention experiments in wild type mice, which administered either Pscr or pSirt1‐4, combined with D‐gal. We performed HE, P21, and SA‐β‐gal staining and observed that P21‐positive cells and relative SA‐β‐gal activity significantly decreased after peptide treatment (Figure 6K–N). Serum biochemical analysis revealed no significant alterations in AST, UREA, or CREA levels, indicating that the peptide caused no overt toxicity and did not induce apparent hepatic or renal injury in mice (Figure S6G–I). Taken together, these results suggest that blocking USP25 from interacting with SIRT1 to increase SIRT1 deacetylase activity via pSirt1‐4 ameliorates aging‐related phenotypes.

## 3 Discussion

In the present study, we demonstrated that USP25 significantly improved cellular senescence and age‐related phenotypes both in vitro and in vivo. We established a mechanistic model in which USP25 directly interacts with SIRT1 and, by specifically removing K63‐linked ubiquitin chains, modulates its deacetylase activity, leading to increased acetylation of P53 and PGC1α. The pSirt1‐4 peptide disrupted the interaction between USP25 and SIRT1, increased SIRT1 deacetylase activity, and ultimately ameliorated cell senescence (Figure 6O). While USP22, which regulates SIRT1 stability, has been identified (Lin et al. 2012), the precise mechanism by which SIRT1 ubiquitination regulates its activity is not fully understood. Our findings reveal that the USP25–SIRT1 regulatory axis is a promising therapeutic target for treating senescence‐related diseases. More importantly, USP25 knockout (KO) does not affect embryonic development or survival in mice, whereas USP22 KO causes embryonic lethality in mice. This advantage over targeting USP22 is a critical prerequisite for the development of USP25 as a therapeutic target and lays the foundation for USP25‐based antiaging interventions.

As a DUB family member, USP25 has been predominantly investigated in the contexts of intestinal homeostasis, tumorigenesis, DNA damage repair, and neurodegeneration. USP25 restricts intestinal pathogen colonization by modulating the expression of colonic proinflammatory cytokines and antimicrobial genes and inhibits colorectal cancer progression by suppressing Wnt signaling and fine‐tuning the SOCS3–Stat3 axis (Wang et al. 2020; Xu et al. 2017). In hepatocellular carcinoma (HCC) cells, USP25 interacts with TRIM21 to regulate the Wnt/β‐catenin pathway and the tumor microenvironment, thereby promoting cancer cell proliferation and migration (Liu et al. 2023). Additionally, USP25 promotes NHEJ repair by deubiquitinating SHLD2, leading to reduced chemosensitivity in cancer cells (Li et al. 2024), and it participates in neurodegenerative processes by regulating microglial homeostasis and neuroinflammation (Zheng et al. 2021, 2022). Despite these advances, whether USP25 contributes to the regulation of organismal aging and its functional role in senescence‐related pathways and underlying molecular mechanisms remain unexplored. In the current study, we demonstrated that USP25 is a pivotal regulator of organismal aging. USP25 knockout alleviated age‐related phenotypes and suppressed cellular senescence both in vitro and in vivo. Mechanistically, USP25 accelerates cellular senescence through the deubiquitination of the lysine residues K506 and K513 of SIRT1. Overall, our results indicate that USP25 is a potential therapeutic target for treating senescence because of its ability to modulate SIRT1 activity.

Although current antiaging strategies, including senolytic, senomorphic, and stem cell therapies, as well as novel agents such as resveratrol, curcumin, and NAD^+^ precursors‐have shown promise in animal models, they are hindered by nonspecific targeting and poor clinical translatability, underscoring the urgent need to identify the precise mechanisms regulating aging and clinically applicable intervention tools (de Magalhães 2024; Guarente et al. 2024). Peptide‐based therapeutics offer distinct advantages, including a low molecular weight, high target affinity, tunable chemical modification, and low immunogenicity, which effectively circumvent the limitations of traditional small‐molecule compounds (e.g., off‐target effects and nonspecificity) (Fosgerau and Hoffmann 2015; Muttenthaler et al. 2021; Wang et al. 2022). In this study, we designed and identified a novel peptide (pSirt1‐4) through molecular modeling that targeted the USP25–SIRT1 interaction interface. Functional validation assays confirmed that pSirt1‐4 specifically disrupted the USP25–SIRT1 complex, increased SIRT1 deacetylase activity, and effectively mitigated doxorubicin‐induced cell senescence, highlighting its potential as a novel antiaging intervention strategy. Consistent with these in vitro findings, pSirt1‐4 treatment ameliorated aging‐related phenotypes in vivo, including alleviating histopathological changes, with no obvious hepatic or renal toxicity. In conclusion, our findings identified a novel peptide, pSirt1‐4, targeting the USP25–SIRT1 interaction interface. This peptide specifically disrupts the USP25–SIRT1 complex, increases SIRT1 deacetylase activity, and effectively alleviates aging‐related phenotypes both in vitro and in vivo, demonstrating its potential as a precision medicine strategy for anti‐aging.

### 3.1 Limitations of the Study

Although we have preliminarily demonstrated, through pharmacological inhibition of SIRT1 using the SIRT1 inhibitor EX527, that the anti‐aging effects mediated by USP25 deficiency depend, at least in part, on SIRT1 activity, it must be noted that pharmacological approaches have inherent limitations and cannot fully recapitulate the persistence and tissue specificity of genetic deletion. Ideally, the generation of *Usp25/Sirt1* double‐knockout mice would provide the most compelling genetic support for a causal relationship within this axis. However, germline deletion of *Sirt1* in mice results in severe developmental defects and perinatal lethality, making the conventional generation of *Usp25/Sirt1* double‐knockout mice technically challenging. Therefore, this validation remains an important goal for future studies and may need to be achieved through alternative strategies such as conditional knockout, tissue‐specific Cre lines, or inducible gene editing.

In addition, given the established functions of USP25 in regulating inflammation and immune responses, although we observed in *Usp25* knockout mice that the mRNA levels of Senescence‐Associated Secretory Phenotype (SASP)‐related factors with immunoregulatory functions were significantly downregulated in D‐gal‐treated mice, and that EX527 treatment reversed this suppression—indicating that direct regulation via the USP25–SIRT1 axis contributes to the improvement in the aging phenotype observed in vivo. However, we can't entirely exclude the possibility that indirect effects of USP25 deficiency on the immune system contribute in part to the amelioration of systemic inflammation, nor that USP25 regulates aging through biological processes beyond the USP25–SIRT1–p53 axis. Future studies using tissue‐specific knockout mice will help to distinguish the functions of USP25 across different cell types and tissues, thereby more precisely defining its direct mechanism of action in aging regulation.

Although *Usp25* ^−/−^ mice exhibit a significantly longer lifespan and improved motor function, and USP25 expression increases with age in naturally aged mice, this evidence is still insufficient to fully establish that USP25 plays an important role in natural aging. The current study is primarily based on knockout models and a limited number of age time points, and developmental compensation effects or specific experimental conditions cannot yet be excluded. Therefore, future systematic studies employing conditional *Usp25/Sirt1* double‐knockout mice under natural aging conditions and at multiple time points, along with clarification of their differential roles across distinct aging stages and pathological contexts, will help to more comprehensively assess the translational potential of the USP25–SIRT1 signaling axis as an anti‐aging target.

## 4 Methods and Materials

### 4.1 Animal Models

All mouse experimental protocols were reviewed and authorized by the Animal Experiment Committee of Tongji University (approval no: TJBB01924101). The study was conducted in full compliance with the relevant legal provisions, regulatory requirements, animal care and maintenance standards, and basic guidelines for animal research. *Usp25* ^−/−^ mice and their wild‐type control littermates (*Usp25* ^+^/^+^) were generated by crossing *Usp25* ^+^/^−^ heterozygous mice. All experiments were conducted using littermate controls for comparison. To establish the aging model, the *Usp25* ^+/+^ and *Usp25* ^−/−^ mice were randomly divided into two groups when they reached approximately 5 months of age. The mice of both genotypes received daily intraperitoneal injections of D‐galactose (100 mg/kg body weight) for 8 consecutive weeks, while the corresponding control groups were administered an equal volume of sterile normal saline via the same route and schedule.

### 4.2 Rotarod Test

The rotarod test was performed to assess motor coordination and fatigue in the mice. Prior to the experiment, the rotarod apparatus was preheated and configured to rotate at speeds ranging from 5 to 30 rpm for a total test duration of 30 min. Health‐matched mice were acclimatized to the laboratory environment for 1–2 days before the experiment. During a pretraining session, the mice were gently placed on the rod and rotated at 5 rpm for 3–5 min to allow them to adapt to the apparatus. In the formal test, each mouse was placed on the rotarod operating at a constant speed of 20 rpm, and the latency to fall was recorded as a measure of motor fatigue. Each mouse underwent three trials with sufficient rest intervals between tests. The average fall latency from three trials was calculated for each mouse, and the means of each group were compared using appropriate statistical methods to evaluate motor performance.

### 4.3 Cell Lines

HEK‐293 T cells were obtained from the American Type Culture Collection (ATCC). The cells were maintained in DMEM supplemented with 10% fetal bovine serum (FBS) and penicillin–streptomycin at 37°C in a 5% CO_2_ humidified atmosphere. The MCF‐10A cell line was cultured under identical conditions in complete MCF‐10A medium.

### 4.4 Coimmunoprecipitation

The cells were scraped, pelleted, and lysed in NETN buffer (100 mM NaCl, 1 mM EDTA, 0.5% NP‐40, 20 mM Tris–HCl [pH 8.0]) supplemented with protease inhibitors for 30 min at 4°C. After centrifugation, 80 μL of the supernatant was reserved as the whole‐cell lysate. The remaining supernatant was incubated with tag‐specific antibody‐conjugated agarose beads for 2–4 h at 4°C. The beads were then washed five times with NETN buffer, and bound proteins were eluted with 1× SDS loading buffer by boiling. Input and co‐IP samples were analyzed by immunoblotting.

### 4.5 Western Blotting

Protein samples were denatured at 100°C for 5 min and centrifuged at 12,000×*g* for 1 min. The proteins were separated by SDS–PAGE (7.5%–15% gel) at 80 V for 10 min, followed by 120 V for 30–40 min. The proteins were transferred to a PVDF membrane activated in methanol using a semidry system at 1.3 A for 10 min. The membrane was blocked with 5% nonfat milk for 1 h at room temperature and then incubated with primary antibody overnight at 4°C. After being washed with TBST, the membrane was incubated with an HRP‐conjugated secondary antibody for 1 h at room temperature. The protein bands were visualized using ECL reagent.

### 4.6 Sample Collection and RNA Sequencing

After the experimental animals were sacrificed, liver tissue samples were collected immediately and rapidly frozen in liquid nitrogen to preserve RNA integrity. Total RNA extraction, sequencing library construction, and sequencing experiments were performed by Majorbio Bio‐Pharm Technology Co. Ltd. (Shanghai, China).

### 4.7 Gene Set Enrichment Analysis (GSEA)

The collected mouse liver samples were snap‐frozen in liquid nitrogen and stored until transcriptome sequencing was performed. The transcriptome sequencing data were analyzed using the free online cloud platform Majorbio Cloud (https://cloud.majorbio.com/, Shanghai, China). The Molecular Signatures Database (MSigDB) was utilized to screen signature gene sets, and the mechanisms of USP25 in aging regulation were analyzed on the basis of the differentially expressed genes in the transcriptome.

### 4.8 RNA Extraction and RT‐qPCR

Total RNA was extracted from cultured cells using an RNA Extraction Kit (ABclonal, RK30120) according to the manufacturer's instructions. The RNA concentration and purity were determined spectrophotometrically. cDNA was synthesized from 1 μg of total RNA using a reverse transcription kit (AG11728; Aikerui Bio). Quantitative PCR was performed using SYBR Green qPCR Master Mix (AG11734; Aikerui Bio) on a QuantStudio 6 Flex system (Applied Biosystems). The thermal cycling conditions were as follows: initial denaturation at 95°C for 30 s, followed by 40 cycles of 95°C for 5 s and 60°C for 30 s. Gene expression levels were normalized to those of GAPDH, and the relative quantification was performed using the 2^−ΔΔCt^ method. All reactions were performed in triplicate.

The sequences of the primers used were as follows:

- *USP25* (*human*): Forward 5′‐AGCACTTCCTGGCAATGATAGA‐3′,
- Reverse 5′‐CCTCCAAACTTCTGTAGGTGTC‐3′;
- *Usp25* (*mouse*): Forward 5′‐GGAGGAGACAGGCTATTACCA‐3′,
- Reverse 5′‐TCAAGGCAATCGCTCTCTGAA‐3′;
- *Gapdh* (*mouse*): Forward 5′‐TTCCAGTATGACTCCACTCACG‐3′,
- Reverse 5′‐AAGACACCAGTAGACTCCACGA‐3′;
- *GAPDH* (*human*): Forward 5′‐CACAAGAGGAAGAGAGAGACC‐3′,
- Reverse 5′‐CCTCTTCAAGGGGTCTACAT‐3′;
- *P21* (*mouse*): Forward 5′‐CCTGGTGATGTCCGACCTG‐3′,
- Reverse 5′‐CCATGAGCGCATCGCAATC‐3′; and
- *P16* (*mouse*): Forward 5′‐CGCAGGTTCTTGGTCACTGT‐3′,
- Reverse 5′‐TGTTCACGAAAGCCAGAGCG‐3′.

### 4.9 Plasmids and shRNAs

The coding sequences of USP25^WT^ and USP25^C178S^ were inserted into the PLVX‐CMV‐HA vector. Full‐length and truncated versions of SIRT1 were constructed using the PLVX‐CMV‐Myc plasmid or PLVX‐CMV‐Flag plasmid. All point mutations were generated using site‐directed mutagenesis (Stratagene). USP25‐targeting shRNAs with the following sequences were obtained from Sigma‐Aldrich:

- shUSP25#1: GCTGTAGAAGATATGAGAAAT (TRCN0000004367); and
- shUSP25#2: GCGTGAGCTGAGGTATCTATT (TRCN0000004369).

### 4.10 Antibodies and Drugs

The following drugs were used in this study: D‐(+)‐Galactose (ST1218; Beyotime), EX527 (SC0281; Beyotime), TSA (T129665; Aladdin), and doxorubicin (HY‐15142R; MCE). The concentrations used for each treatment are indicated in the corresponding figure legends.

The primary antibodies used in this study were as follows: anti‐USP25 (Abcam: ab187156, WB: 1:1000; Proteintech: 12199‐1‐AP, IHC: 1:250), anti‐Ub (Santa Cruz: sc‐8017, WB: 1:1000), anti‐His (Proteintech: 10001‐0‐AP, WB: 1:500), anti‐Tubulin (Proteintech: 66031‐1‐lg, WB: 1:1000), anti‐GAPDH (ABclonal: A19056, WB: 1:1000), anti‐β‐actin (Sigma: A2228, WB: 1:2000), anti‐Flag (Proteintech: 20543‐1‐AP, WB: 1:1000), anti‐HA (ABclonal: AE036, WB: 1:2000), and anti‐Myc (Proteintech: 16286‐1‐AP, WB: 1:1000), anti‐SIRT1 (Proteintech: 13161‐1‐AP, WB: 1:1000), Ace‐P53(Lys382) (CST: 2525, WB: 1:1000), P53 (Proteintech: 10442‐1‐AP, WB: 1:1000), DBC1 (ABclonal: A24534, WB: 1:1000), anti‐P21 (Servicebio: GB15153‐50, WB: 1:1000), and Pan‐ace (ptm‐biolab: PTM‐102, WB: 1:1000).

### 4.11 Peptide Synthesis and Treatment

All the peptides were chemically synthesized by SBS Genetech Co. Ltd. (Beijing, China), each with a purity exceeding 95%, as verified by high‐performance liquid chromatography (HPLC). The synthesized products were suitable for use in both in vitro and in vivo assays. The amino acid sequences of the peptides pSirt1‐1, pSirt1‐2, pSirt1‐3, and pSirt1‐4 are as follows:

- pSirt1‐1: HLYVSPWRLGGEYAKLCCNPVK
- pSirt1‐2: HLYVSPWCCNPVKLSEITEKPP
- pSirt1‐3: HLYVSPWEQMENPDLKNVGSST
- pSirt1‐4: HLYVSPWWPNRVAKEQISRRLD

### 4.12 IHC Staining and HE Staining

For immunohistochemical staining, tissue sections were first deparaffinized in xylene and rehydrated through a graded ethanol series, followed by a 5 min wash with PBS. Antigen retrieval was performed using a specific antigen repair solution. The sections were then blocked with 5% FBS for 2 h at room temperature. Primary antibody incubation was carried out overnight at 4°C, followed by 20 min of equilibration at room temperature. After three washes with PBS containing 0.1% Triton X‐100, the sections were incubated with a secondary antibody for 2 h at 25°C. Unbound secondary antibodies were removed by washing three times with PBS containing 0.25% Triton X‐100. DAB substrate was applied for color development, and the reaction was terminated by the addition of PBS. All the results, including staining intensity and percentage of positive cells, were evaluated by pathologists who were blinded to the groupings. For HE staining, tissue sections were deparaffinized in xylene and rehydrated through graded ethanol. After being rinsed in distilled water, the sections were stained with hematoxylin for 5 min, washed, differentiated in 1% acid alcohol, and counterstained with eosin for 2 min. Dehydration was performed through an ethanol series and xylene.

### 4.13 Deubiquitination Assay

For the deubiquitination assay, USP25‐knockdown cells were cultured to ≈50% confluence in 10 cm dishes and cotransfected with the HA‐Ub plasmid and Flag‐SIRT1 plasmid. After 8 h of incubation, the medium was replaced. The cells were then fully lysed in NETN buffer containing a protease inhibitor cocktail, and the lysates were clarified by centrifugation at 12,000×*g* for 15 min at 4°C to obtain the supernatants. Flag‐tag‐specific immunoprecipitation was performed on the whole‐cell extracts, followed by multiple washes to eliminate nonspecifically bound proteins, after which the proteins were subjected to immunoblotting analysis for detection.

### 4.14 Measurement of SIRT1 Deacetylase Activity

SIRT1 deacetylase activity in HEK‐293 T and MCF‐10A cells was quantified using a SIRT1 deacetylase activity assay kit (E‐BC‐F056; Elabscience). Cells (1 × 10^6^ per sample) were homogenized in 0.2 mL of physiological saline (0.9% NaCl) and centrifuged at 10,000×*g* for 10 min at 4°C. The supernatant was retained for immediate analysis, and the total protein concentration was determined using a BCA assay kit (Beyotime, P0010S). All the reagents were equilibrated to room temperature before use. The reaction working solution was prepared by mixing buffer (Reagent 1), substrate (Reagent 2), and reconstituted accelerant (Reagent 3) at a volume ratio of 100:1:2. A standard curve was generated from the data acquired with the 1 mmol/L standard (Reagent 5) diluted to 0–100 μmol/L. Each 5 μL sample, standard, or saline blank was added to duplicate wells of a black 96‐well plate, followed by 100 μL of reaction working solution (for samples and blank) or buffer (for standards). After mixing for 3 s, the plate was incubated at 37°C for 20 min. Then, 20 μL of activator (Reagent 4) was added to each well, and the samples were mixed and incubated at 37°C for an additional 15 min. Fluorescence was measured at 340/440 nm. SIRT1 deacetylase activity (U/g prot) was calculated on the basis of the standard curve (*y* = *ax* + *b*) and the following formula:

$$\text{SIRT}‐1\;\text{Activity}=\frac{\left(\Delta F-b\right)}{a\times T\times C_{\mathrm{pr}}}\times f$$

where Δ*F* represents sample fluorescence minus that of the blank; *a* is the slope of the calibration curve; *b* is the *y*‐intercept of the calibration curve; *T* = 20 min; *C*_pr_ denotes the sample protein concentration (g prot/L); and *f* is the dilution factor.

### 4.15 Protein Quantification

The total protein concentration in the cell lysates was quantified using a bicinchoninic acid (BCA) protein assay kit (Beyotime) in strict accordance with the manufacturer's protocol to ensure accuracy. Briefly, bovine serum albumin (BSA) standards were serially diluted (0, 250, 500, 750, 1000, 1500, and 2000 μg/mL) to generate a linear standard curve, covering the full dynamic range of the assay. The cell lysate samples were appropriately diluted with sterile PBS (pH 7.4) to ensure that their protein concentrations fell within the linear range of the standard curve (250–2000 μg/mL). Equal volumes (20 μL) of BSA standards and diluted samples were added to a 96‐well microplate in duplicate, after which 200 μL of freshly prepared BCA working reagent (Reagent A:Reagent B = 50:1, v/v) was added to each well. The plate was gently shaken to thoroughly mix the samples and incubated at 37°C for 30 min in the dark to prevent light‐induced interference. After incubation, the absorbance of each well was immediately measured at 562 nm using a microplate reader (BioTek, Synergy H1), with blank wells (containing only the BCA working reagent and PBS) used for background subtraction. The protein concentrations of the samples were calculated from the standard curve using linear regression analysis and adjusted for the corresponding dilution factors to obtain the final concentration in the undiluted lysates. All measurements were performed in technical duplicates to minimize experimental variability, and average values were used for subsequent experiments.

### 4.16 SA‐β‐Gal Staining

Cellular senescence was assessed using a Senescence β‐Galactosidase Staining Kit (#9860, Cell Signaling Technology, Beverly, MA, USA) according to the manufacturer's protocol. MCF‐10A cells were seeded in 6‐well plates and cultured until they reached the appropriate confluency. After the growth medium was removed, the cells were gently washed once with 1× PBS (2 mL per well for 35 mm dishes). Afterwards, 1 mL of 1× fixative solution was added to each well and incubated at room temperature for 10–15 min. Following fixation, the cells were washed twice with 1× PBS. The plates were then stored in PBS at 4°C overnight if needed. Afterwards, 1 mL of β‐galactosidase staining solution was added to each well. To prevent evaporation and crystal formation, the plates were sealed with parafilm and incubated in a dry, CO_2_‐free incubator at 37°C for at least 12–16 h. The absence of CO_2_ is critical for maintaining a proper pH for activity. Cells were examined under a light microscope at 200× total magnification to allow the development of blue staining, which is indicative of SA‐β‐gal activity.

### 4.17 Statistical Analysis

Statistical analyses were performed using Prism 8.0 software (GraphPad). We used the two‐tailed Student's *t*‐tests for comparisons between two groups and one‐way ANOVA or two‐way ANOVA for comparisons among three or more groups. *p* < 0.05 was considered to indicate statistical significance. *p < 0.05, **p < 0.01, ***p < 0.001, ****p < 0.0001; ns not significant. All data were presented as means ± SD. Sample sizes are included in the figure legends.

## Author Contributions

YuJuan Zhou, Xinshu Wang, and Jiyuan Zhang contributed equally to this work. Yunhui Li, Lei Li, and Jian Yuan conceived and designed the study. YuJuan Zhou and Xinshu Wang performed the experiments, analyzed the data, and drafted the manuscript. Jiyuan Zhang, Hanqiong Qiang, Xinru Zhao, and Jiakai Yao established the mouse model and conducted related experiments. Ke Li and Tingting Zhang conducted the peptide experiment. Jingyi Luo and Yanjun Ji performed digital scanning of the IHC‐stained slides. Linhui Zhai conducted the bioinformatics data. Yuping Chen provided the constructional suggestions for this paper. Yunhui Li, Lei Li, and Jian Yuan edited and revised the manuscript. All the authors provided constructive suggestions and assistance for this article. All the authors have read and approved the final version of the manuscript to be published.

## Funding

This research was supported by funding from the National Key Research and Development Plan (Grant No. 2022YFA1302803), the Fundamental Research Funds for the Central Universities (Grant Nos. 22120240382 and 22120260447), the Tongji University Medicine‐X Interdisciplinary Research Initiative (2025‐0554‐ZD‐05), and the Shanghai Tongji University Education Development Foundation.

## Ethics Statement

This study strictly adhered to all applicable ethical standards for animal research. The *USP25* ^−/−^ mice utilized in the experiments were generated by Shanghai Model Organisms via CRISPR/Cas9 technology. All animal‐related procedures were conducted in full compliance with the experimental protocols approved by the Shanghai Model Organisms Center Inc. (approval numbers: 2019‐0026; 2022‐0030).

## Conflicts of Interest

The authors declare no conflicts of interest.

## Supporting information


**Figure S1:** USP25 deficiency ameliorates senescence in the natural aging mouse model.
**Figure S2:** USP25 deficiency ameliorates senescence in the D‐gal‐induced mouse model.
**Figure S3:** USP25 directly deubiquitinates SIRT1 in vivo.
**Figure S4:** USP25 deletion suppresses cell senescence through SIRT1‐mediated P53 acetylation.
**Figure S5:** USP25 deubiquitinates SIRT1 at K506/K513 and modulates its activity through DBC1.
**Figure S6:** Blocking the USP25–SIRT1 interaction by peptide competition enhances SIRT1 activity.

## Data Availability

Data sharing not applicable to this article as no datasets were generated or analyzed during the current study.

## References

[acel70786-bib-0001] Baker, D. J. , B. G. Childs , M. Durik , et al. 2016. “Naturally Occurring p16(Ink4a)‐Positive Cells Shorten Healthy Lifespan.” Nature 530, no. 7589: 184–189. 10.1038/nature16932. PMC4845101 26840489

[acel70786-bib-0002] Buetow, L. , and D. T. Huang . 2016. “Structural Insights Into the Catalysis and Regulation of E3 Ubiquitin Ligases.” Nature Reviews Molecular Cell Biology 17, no. 10: 626–642. 10.1038/nrm.2016.91. PMC6211636 27485899

[acel70786-bib-0003] Cai, C. , H. Ma , J. Peng , et al. 2023. “USP25 Regulates KEAP1‐NRF2 Anti‐Oxidation Axis and Its Inactivation Protects Acetaminophen‐Induced Liver Injury in Male Mice.” Nature Communications 14, no. 1: 3648. 10.1038/s41467-023-39412-6. PMC10282087 37339955

[acel70786-bib-0004] Chau, V. , J. W. Tobias , A. Bachmair , et al. 1989. “A Multiubiquitin Chain Is Confined to Specific Lysine in a Targeted Short‐Lived Protein.” Science 243, no. 4898: 1576–1583. 10.1126/science.2538923. 2538923

[acel70786-bib-0005] Childs, B. G. , M. Durik , D. J. Baker , et al. 2015. “Cellular Senescence in Ageing and Age‐Related Disease: From Mechanisms to Therapy.” Nature Medicine 21, no. 12: 1424–1435. 10.1038/nm.4000. PMC4748967 26646499

[acel70786-bib-0006] Chini, E. N. , C. C. S. Chini , V. Nin , et al. 2013. “Deleted in Breast Cancer‐1 (DBC‐1) in the Interface Between Metabolism, Ageing and Cancer.” Bioscience Reports 33, no. 4: e00058. 10.1042/BSR20130062. PMC3755336 23841676

[acel70786-bib-0007] Coppé, J. P. , C. K. Patil , F. Rodier , et al. 2008. “Senescence‐Associated Secretory Phenotypes Reveal Cell‐Nonautonomous Functions of Oncogenic RAS and the p53 Tumor Suppressor.” PLoS Biology 6, no. 12: 2853–2868. 10.1371/journal.pbio.0060301. PMC2592359 19053174

[acel70786-bib-0008] Cunningham, C. N. , J. M. Baughman , L. Phu , et al. 2015. “USP30 and Parkin Homeostatically Regulate Atypical Ubiquitin Chains on Mitochondria.” Nature Cell Biology 17, no. 2: 160–169. 10.1038/ncb3097. 25621951

[acel70786-bib-0009] Dammer, E. B. , C. H. Na , P. Xu , et al. 2011. “Polyubiquitin Linkage Profiles in Three Models of Proteolytic Stress Suggest the Etiology of Alzheimer Disease.” Journal of Biological Chemistry 286, no. 12: 10457–10465. 10.1074/jbc.M110.149633. PMC3060499 21278249

[acel70786-bib-0010] de Magalhães, J. P. 2024. “Cellular Senescence in Normal Physiology.” Science 384, no. 6702: 1300–1301. 10.1126/science.adj7050. 38900869

[acel70786-bib-0011] Di Micco, R. , V. Krizhanovsky , D. Baker , et al. 2021. “Cellular Senescence in Ageing: From Mechanisms to Therapeutic Opportunities.” Nature Reviews Molecular Cell Biology 22, no. 2: 75–95. 10.1038/s41580-020-00314-w. PMC8344376 33328614

[acel70786-bib-0012] Dikic, I. , and B. A. Schulman . 2023. “An Expanded Lexicon for the Ubiquitin Code.” Nature Reviews Molecular Cell Biology 24, no. 4: 273–287. 10.1038/s41580-022-00543-1. PMC9595094 36284179

[acel70786-bib-0013] Dimri, G. P. , X. Lee , G. Basile , et al. 1995. “A Biomarker That Identifies Senescent Human Cells in Culture and in Ag Ing Skin In Vivo.” Proceedings of the National Academy of Sciences of the United States of America 92, no. 20: 9363–9367. 10.1073/pnas.92.20.9363. PMC40985 7568133

[acel70786-bib-0014] Dulić, V. , G. E. Beney , G. Frebourg , et al. 2000. “Uncoupling Between Phenotypic Senescence and Cell Cycle Arrest in Ageing p21‐Deficient Fibroblasts.” Molecular and Cellular Biology 20, no. 18: 6741–6754. 10.1128/mcb.20.18.6741-6754.2000. PMC86196 10958672

[acel70786-bib-0015] Fosgerau, K. , and T. Hoffmann . 2015. “Peptide Therapeutics: Current Status and Future Directions.” Drug Discovery Today 20, no. 1: 122–128. 10.1016/j.drudis.2014.10.003. 25450771

[acel70786-bib-0016] Goldbraikh, D. , D. Neufeld , Y. Eid‐Mutlak , et al. 2020. “USP1 Deubiquitinates Akt to Inhibit PI3K‐Akt‐FoxO Signaling in Muscle During Prolonged Starvation.” EMBO Reports 21, no. 4: e48791. 10.15252/embr.201948791. PMC7132338 32133736

[acel70786-bib-0017] Guarente, L. , D. A. Sinclair , and G. Kroemer . 2024. “Human Trials Exploring Anti‐Ageing Medicines.” Cell Metabolism 36, no. 2: 354–376. 10.1016/j.cmet.2023.12.007. 38181790

[acel70786-bib-0018] Hernandez‐Segura, A. , J. Nehme , and M. Demaria . 2018. “Hallmarks of Cellular Senescence.” Trends in Cell Biology 28, no. 6: 436–453. 10.1016/j.tcb.2018.02.001. 29477613

[acel70786-bib-0020] Kang, H. , J. Y. Suh , Y. S. Jung , J. W. Jung , M. K. Kim , and J. H. Chung . 2011. “Peptide Switch Is Essential for Sirt1 Deacetylase Activity.” Molecular Cell 44, no. 2: 203–213. 10.1016/j.molcel.2011.07.038. PMC3240942 22017869

[acel70786-bib-0021] Kim, J. E. , J. J. Chen , and Z. K. Lou . 2008. “DBC1 Is a Negative Regulator of SIRT1.” Nature 451, no. 7178: 583–586. 10.1038/nature06500. 18235501

[acel70786-bib-0022] Komander, D. , M. J. Clague , and S. Urbé . 2009. “Breaking the Chains: Structure and Function of the Deubiquitinases.” Nature Reviews Molecular Cell Biology 10, no. 8: 550–563. 10.1038/nrm2731. 19626045

[acel70786-bib-0023] Komander, D. , and M. Rape . 2012. “The Ubiquitin Code.” Annual Review of Biochemistry 81: 203–229. 10.1146/annurev-biochem-060310-170328. 22524316

[acel70786-bib-0024] Kumari, R. , and P. Jat . 2021. “Mechanisms of Cellular Senescence: Cell Cycle Arrest and Senescence as Sociated Secretory Phenotype.” Frontiers in Cell and Developmental Biology 9: 645593. 10.3389/fcell.2021.645593. PMC8039141 33855023

[acel70786-bib-0025] Kumari, R. , R. Kumar , S. Kumar , et al. 2020. “Amyloid Aggregates of the Deubiquitinase OTUB1 Are Neurotoxic, Suggesting That They Contribute to the Development of Parkinson's Disease.” Journal of Biological Chemistry 295, no. 11: 3466–3484. 10.1074/jbc.RA119.009546. PMC7076217 32005664

[acel70786-bib-0026] Li, J. , Y. Wang , Y. Luo , et al. 2022. “USP5‐Beclin 1 Axis Overrides p53‐Dependent Senescence and Drives Kras‐Induced Tumorigenicity.” Nature Communications 13, no. 1: 7799. 10.1038/s41467-022-35557-y. PMC9759531 36528652

[acel70786-bib-0027] Li, Y. , L. Li , X. Wang , et al. 2024. “USP25 Elevates SHLD2‐Mediated DNA Double‐Strand Break Repair and Regulates Chemoresponse in Cancer.” Advanced Science (Weinh) 11, no. 28: e2403485. 10.1002/advs.202403485. PMC11267380 38803048

[acel70786-bib-0028] Lin, Z. H. , H. Yang , Q. F. Kong , et al. 2012. “USP22 Antagonizes p53 Transcriptional Activation by Deubiquitinating Sirt1 to Suppress Cell Apoptosis and Is Required for Mouse Embryonic Development.” Molecular Cell 46, no. 4: 484–494. 10.1016/j.molcel.2012.03.024. 22542455

[acel70786-bib-0029] Liu, Y. H. , J. J. Ma , S. M. Lu , et al. 2023. “USP25 Promotes Hepatocellular Carcinoma Progression by Interacting With TRIM21 via the Wnt/β‐Catenin Signaling Pathway.” Chinese Medical Journal 136, no. 18: 2229–2242. 10.1097/Cm9.0000000000002714. PMC10508383 37439386

[acel70786-bib-0031] Luo, J. , F. Su , D. Chen , A. Shiloh , and W. Gu . 2000. “Deacetylation of p53 Modulates Its Effect on Cell Growth and Apoptosis.” Nature 408, no. 6810: 377–381. 10.1038/35042612. 11099047

[acel70786-bib-0032] Muñoz‐Espín, D. , and M. Serrano . 2014. “Cellular Senescence: From Physiology to Pathology.” Nature Reviews Molecular Cell Biology 15, no. 7: 482–496. 10.1038/nrm3823. 24954210

[acel70786-bib-0033] Muttenthaler, M. , G. F. King , D. J. Adams , and P. F. Alewood . 2021. “Trends in Peptide Drug Discovery.” Nature Reviews Drug Discovery 20, no. 4: 309–325. 10.1038/s41573-020-00135-8. 33536635

[acel70786-bib-0034] Nacarelli, T. , L. Lau , T. Fukumoto , et al. 2019. “NAD(+) Metabolism Governs the Proinflammatory Senescence‐Associated Secretome.” Nature Cell Biology 21, no. 3: 397–407. 10.1038/s41556-019-0287-4. PMC6448588 30778219

[acel70786-bib-0035] Nijman, S. M. B. , M. P. A. Luna‐Vargas , A. Velds , et al. 2005. “A Genomic and Functional Inventory of Deubiquitinating Enzymes.” Cell 123, no. 5: 773–786. 10.1016/j.cell.2005.11.007. 16325574

[acel70786-bib-0036] Park, J.‐S. , H. Ma , and Y.‐S. Roh . 2021. “Ubiquitin Pathways Regulate the Pathogenesis of Chronic Liver Disease.” Biochemical Pharmacology 193: 114764. 10.1016/j.bcp.2021.114764. 34529948

[acel70786-bib-0037] Petrova, N. V. , A. K. Velichko , S. V. Razin , and O. L. Kantidze . 2016. “Small Molecule Compounds That Induce Cellular Senescence.” Aging Cell 15, no. 6: 999–1017. 10.1111/acel.12518. PMC6398529 27628712

[acel70786-bib-0038] Rossiello, F. , U. Herbig , M. P. Longhese , M. Fumagalli , and F. d'Adda di Fagagna . 2014. “Irreparable Telomeric DNA Damage and Persistent DDR Signalling as a Shared Causative Mechanism of Cellular Senescence and Ageing.” Current Opinion in Genetics & Development 26: 89–95. 10.1016/j.gde.2014.06.009. PMC4217147 25104620

[acel70786-bib-0039] Sahu, Y. , P. Jamadade , K. Ch Maharana , and S. Singh . 2024. “Role of Mitochondrial Homeostasis in D‐Galactose‐Induced Cardiovascular Ageing From Bench to Bedside.” Mitochondrion 78: 101923. 10.1016/j.mito.2024.101923. 38925493

[acel70786-bib-0040] Swatek, K. N. , and D. Komander . 2016. “Ubiquitin Modifications.” Cell Research 26, no. 4: 399–422. 10.1038/cr.2016.39. PMC4822133 27012465

[acel70786-bib-0041] Ungurianu, A. , A. Zanfirescu , and D. Margină . 2023. “Sirtuins, Resveratrol and the Intertwining Cellular Pathways Connecting Them.” Ageing Research Reviews 88: 101936. 10.1016/j.arr.2023.101936. 37116286

[acel70786-bib-0042] Valero, R. , G. Marfany , O. González‐Angulo , G. González‐González , L. Puelles , and R. Gonzàlez‐Duarte . 1999. “USP25, a Novel Gene Encoding a Deubiquitinating Enzyme, Is Located in the Gene‐Poor Region 21q11.2.” Genomics 62, no. 3: 395–405. 10.1006/geno.1999.6025. 10644437

[acel70786-bib-0043] van der Horst, A. , L. G. Tertoolen , L. M. de Vries‐Smits , et al. 2004. “FOXO4 Is Acetylated Upon Peroxide Stress and Deacetylated by the Longevity Protein hSir2(SIRT1).” Journal of Biological Chemistry 279, no. 28: 28873–28879. 10.1074/jbc.M401138200. 15126506

[acel70786-bib-0044] Vaseenon, S. , N. Apaijai , W. Pratchayasakul , et al. 2025. “D‐Galactose‐Induced Ageing and Obese Conditions Contribute to Ageing and Pathologies in Dental Pulp of Male Wistar Rats.” Experimental Gerontology 211: 112907. 10.1016/j.exger.2025.112907. 40992644

[acel70786-bib-0045] Wang, L. , N. Wang , W. Zhang , et al. 2022. “Therapeutic Peptides: Current Applications and Future Directions.” Signal Transduction and Targeted Therapy 7, no. 1: 48. 10.1038/s41392-022-00904-4. PMC8844085 35165272

[acel70786-bib-0046] Wang, X. M. , C. Yang , Y. Zhao , et al. 2020. “The Deubiquitinase USP25 Supports Colonic Inflammation and Bacterial Infection and Promotes Colorectal Cancer.” Nature Cancer 1, no. 8: 811–825. 10.1038/s43018-020-0089-4. 35122046

[acel70786-bib-0047] Xu, D. , J. Liu , T. Fu , et al. 2017. “USP25 Regulates Wnt Signaling by Controlling the Stability of Tankyrases.” Genes & Development 31, no. 10: 1024–1035. 10.1101/gad.300889.117. PMC5495119 28619731

[acel70786-bib-0048] Xu, M. , T. Pirtskhalava , J. N. Farr , et al. 2018. “Senolytics Improve Physical Function and Increase Lifespan in Old Age.” Nature Medicine 24, no. 8: 1246–1256. 10.1038/s41591-018-0092-9. PMC6082705 29988130

[acel70786-bib-0049] Zhao, W. H. , J. P. Kruse , Y. Tang , et al. 2008. “Negative Regulation of the Deacetylase SIRT1 by DBC1.” Nature 451, no. 7178: 587–590. 10.1038/nature06515. PMC2866287 18235502

[acel70786-bib-0050] Zheng, Q. , B. Song , G. Li , et al. 2022. “USP25 Inhibition Ameliorates Alzheimer's Pathology Through the Regulation of APP Processing and Aβ Generation.” Journal of Clinical Investigation 132, no. 5: e152170. 10.1172/jci152170. PMC8884900 35229730

[acel70786-bib-0051] Zheng, Q. Y. , G. L. Li , S. H. Wang , et al. 2021. “Trisomy 21‐Induced Dysregulation of Microglial Homeostasis in Alzheimer's Brains Is Mediated by USP25.” Science Advances 7, no. 1: eabe1340. 10.1126/sciadv.abe1340. PMC7775784 33523861

[acel70786-bib-0052] Zhou, L. , B. Qin , D. M. Yassine , et al. 2023. “Structure and Function of the Highly Homologous Deubiquitinases Ubiquitin Specific Peptidase 25 and 28: Insights Into Their Pathophysiological and Therapeutic Roles.” Biochemical Pharmacology 213: 115624. 10.1016/j.bcp.2023.115624. 37245535

[acel70786-bib-0053] Zhu, F. Y. , L. L. Wang , T. G. Meng , et al. 2021. “Inhibiting Bridge Integrator 2 Phosphorylation Leads to Improved Oocyte Quality, Ovarian Health and Fertility in Ageing and After Chemotherapy in Mice.” Nature Aging 1, no. 11: 1010–1023. 10.1038/s43587-021-00133-4. 37118338

