# Supplementary material for: A Novel Peptide Targeting the USP25–SIRT1 Interaction Activates SIRT1 to Suppress Cell Senescence
Source: Aging Cell. 2026 Oct 30;25(11):e70786. doi: 10.1111/acel.70786 (PMC13722610; doi:10.1111/acel.70786)
Supplement: Supplementary file 1 [file ACEL-25-e70786-s001.docx]

**Figure S1. USP25 deficiency ameliorates senescence in the natural ageing mouse model.**

**(A)** GSEA of the RNA-sequencing data from 10-month-old *Usp25^+/+^* and *Usp25*^-/-^ mice (n=3 per group).

**(B)** Representative H&E (scale bar=100μm) staining of the lung, brain, heart and kidney tissues of *Usp25^+/+^* and *Usp25*^-/-^ mice.

**(C)** Representative P21 (scale bar=50μm) staining of the lung, brain, heart and kidney tissues of *Usp25^+/+^* and *Usp25*^-/-^ mice.

**(D)** Quantification P21 staining of the lung, brain, heart and kidney tissues of *Usp25^+/+^* and *Usp25*^-/-^ mice. The data are presented as the mean±SD (n=5 per group).

**(E)** Representative images of TRAP and ALP staining in bone sections from *Usp25^+/+^* and *Usp25*^-/-^ mice (scale bar=50μm).

**(F)–(G)** Quantification of **(F)** ALP⁺ osteoblasts and **(G)** TRAP⁺ osteoclasts in *Usp25^+/+^* and *Usp25^−/−^* mice. The data are presented as the mean±SD (n=5 per group).

Statistical analysis was performed by an unpaired *t* test (**D, F and G**), ***p ≤ 0.01*, ****p ≤ 0.001, ****p ≤ 0.0001*.

**Figure S2. USP25 deficiency ameliorates senescence in the D-gal-induced mouse model.**

**(A)** Representative images of TRAP and ALP staining in bone sections from *Usp25^+/+^* and *Usp25^−/−^*^-^ mice between the untreated and after D-gal treatment (scale bar=50μm).

**(B)–(C)** Quantification of **(B)** ALP⁺ osteoblasts and **(C)** TRAP⁺ osteoclasts in *Usp25^+/+^* and *Usp25^−/−^* mice between the untreated and after D-gal treatment. The data are presented as the mean±SD (n=5 per group).

Statistical analysis was performed by two-way ANOVA (**B and C**) . ***p ≤ 0.01*, ****p ≤ 0.001, ****p ≤ 0.0001*.

**Figure S3. USP25 directly deubiquitinates SIRT1 *in vivo.***

**(A)** The protein levels of SIRT1 in lung tissue were determined by immunoblotting.

**(B)** SIRT1 ubiquitination levels were measured in the lung tissue of *Usp25^+/+^* and *Usp25*^-/-^ mice.

**Figure S4. USP25 deletion suppresses cell senescence through SIRT1-mediated P53 acetylation.**

**(A)** Immunoblotting of USP25 in HEK-293T cells stably expressing control or USP25shRNA.

**(B)** ELISA analysis of SIRT1 activity in USP25-knockdown HEK-293T cells. The data are presented as the mean±SD (n=3 per group).

**(C)** ELISA analysis of SIRT1 deacetylase activity in aged *Usp25^+/+^* and *Usp25*^-/-^ mice. The data are presented as the mean±SD (n=3 per group).

**(D)** Western blot analysis of Ace-P53(Lys382) levels in HEK-293T cells with USP25 knockdown, SIRT1 knockdown, or dual USP25 and SIRT1 knockdown cells.

**(E)** Western blot analysis of P53 acetylation in cells cotransfected with GFP-P53 and HA-p300, under conditions of USP25 or SIRT1 knockdown. The blots were probed with the indicated antibodies.

**(F)** Analysis of PGC-1α acetylation levels in USP25-knockdown cells.

**(G)** Western blot analysis of PGC-1α acetylation levels in cells with USP25 knockdown, SIRT1 knockdown, or dual USP25 and SIRT1 knockdown cells.

Statistical analysis was performed via *t* tests (**A**) or one-way ANOVA (**C**), **p ≤ 0.05*, ***p ≤ 0.01*, ****p ≤ 0.001*.

**Figure S5. USP25 deubiquitinates SIRT1 at K506/K513 and modulates its activity through DBC1.**

**(A)** Control or USP25-knockdown HEK-293T cells were transfected with Myc-SIRT1 WT or 2KR mutants.The blots were probed with the indicated antibodies.

**(B)** ELISA analysis of SIRT1 activity in USP25-knockdown HEK-293T cells, transfected with Myc-SIRT or 2KR mutants. The data are presented as the mean±SD (n=3 per group). Statistical significance was determined by two-way ANOVA. *****p ≤ 0.0001*; ns indicates no statistically significant difference.

**(C)** Interaction between DBC1 and SIRT1 in the lung tissue from *Usp25^+/+^* and *Usp25*^-/-^ mice.

**Figure S6. Blocking the USP25–SIRT1 interaction by peptide competition enhances SIRT1 activity.**

**(A)–(D)** The kinetic interaction between the peptides and USP25 was determined by surface plasmon resonance (SPR).

**(E)** The amino acid (aa) sequences of the peptides covering the SIRT1-binding region of USP25 are shown.

**(F)** ELISA analysis of SIRT1 activity was detected after pSirt1-4 treatment in HEK-293T cells. The data are presented as the mean±SD (n=3 per group). Statistical analysis was performed by an unpaired *t* test **p ≤ 0.05*.

**(G)–(I)** Serological testing was conducted to determine the levels of AST, urea, and creatinine (CREA) in mouse serum. Statistical analysis was performed via one-way ANOVA. ns indicates no statistically significant difference.
